# Towards Improved Pharmacokinetic Models for the Analysis of Transporter-Mediated Hepatic Disposition of Drug Molecules with Positron Emission Tomography

**DOI:** 10.1208/s12248-019-0323-0

**Published:** 2019-04-29

**Authors:** Irene Hernández Lozano, Rudolf Karch, Martin Bauer, Matthias Blaickner, Akihiro Matsuda, Beatrix Wulkersdorfer, Marcus Hacker, Markus Zeitlinger, Oliver Langer

**Affiliations:** 1 Department of Clinical Pharmacology, Medical University of Vienna, A-1090 Vienna, Austria; 2 Centre for Medical Statistics, Informatics, and Intelligent Systems, Medical University of Vienna, Vienna, Austria; 3 Preclinical Molecular Imaging, AIT Austrian Institute of Technology GmbH, Seibersdorf, Austria; 4 Division of Nuclear Medicine, Department of Biomedical Imaging and Image-guided Therapy, Medical University of Vienna, Vienna, Austria

**Keywords:** positron emission tomography, hepatobiliary clearance, membrane transporters, pharmacokinetic model, [^11^C]erlotinib

## Abstract

Positron emission tomography (PET) imaging with radiolabeled drugs holds great promise to assess the influence of membrane transporters on hepatobiliary clearance of drugs. To exploit the full potential of PET, quantitative pharmacokinetic models are required. In this study, we evaluated the suitability of different compartment models to describe the hepatic disposition of [^11^C]erlotinib as a small-molecule model drug which undergoes transporter-mediated hepatobiliary excretion. We analyzed two different, previously published data sets in healthy volunteers, in which a baseline [^11^C]erlotinib PET scan was followed by a second PET scan either after oral intake of unlabeled erlotinib (300 mg) or after intravenous infusion of the prototypical organic anion-transporting polypeptide inhibitor rifampicin (600 mg). We assessed a three-compartment (3C) and a four-compartment (4C) model, in which either a sampled arterial blood input function or a mathematically derived dual input function (DIF), which takes the contribution of the portal vein to the liver blood supply into account, was used. Both models provided acceptable fits of the observed PET data in the liver and extrahepatic bile duct and gall bladder. Changes in model outcome parameters between scans were consistent with the involvement of basolateral hepatocyte uptake and canalicular efflux transporters in the hepatobiliary clearance of [^11^C]erlotinib. Our results demonstrated that inclusion of a DIF did not lead to substantial improvements in model fits. The models developed in this work represent a step forward in applying PET as a tool to assess the impact of hepatic transporters on drug disposition and their involvement in drug-drug interactions.

**Electronic supplementary material:**

The online version of this article (10.1208/s12248-019-0323-0) contains supplementary material, which is available to authorized users.

## INTRODUCTION

The liver is the major organ responsible for the metabolism and excretion of xenobiotics. It expresses several different transport proteins belonging to the solute carrier (SLC) and ATP-binding cassette (ABC) families in both the blood-facing basolateral membrane and in the bile-facing canalicular membrane of hepatocytes. These transporters regulate the uptake and biliary secretion of drugs and their metabolites into and out of the hepatocytes and therefore play a key role in the clearance of drugs (1). In addition to drug metabolizing enzymes, transporters can be involved in drug-drug interactions (DDIs). For instance, co-administration of a drug which is a substrate and a drug which is an inhibitor of the same transporter(s) can lead to changes in the pharmacokinetics (PK) of the substrate drug making transporters a potential source of PK variability (2).

The interaction of drugs with transporters and their potential to be involved in DDIs is commonly studied using *in vitro* assays in which, for instance, drug uptake in cell lines overexpressing particular transporters is compared to drug uptake in non-transporter overexpressing control cells. In cases in which *in vitro* data point to a risk for transporter-mediated DDIs, *in vivo* studies in healthy human volunteers may become necessary (3). Usually, transporter-mediated DDIs lead to changes in plasma PK and can be studied *in vivo* by monitoring plasma drug concentrations. However, in other cases, transporter inhibition can lead to pronounced changes in the drug tissue concentrations with a negligible effect on the plasma PK (1,4). In order to assess the influence of transporters on drug tissue distribution, a method to measure drug tissue concentration is needed. Yet, current approaches to determine drug concentration in tissues mostly involve invasive procedures, which are not applicable in humans (5,6).

Noninvasive nuclear imaging methods such as positron emission tomography (PET) or single photon emission computed tomography (SPECT) allow radiolabeled drug molecules in the body to be visualized and monitored, therewith facilitating the study of time-dependent changes in drug tissue concentrations. Accordingly, these imaging methods can be implemented to assess the role of drug transporter function in drug disposition (6,7). In order to fully exploit the potential of these noninvasive imaging approaches in the study of drug transporters, quantitative PK modeling approaches are required. PK modeling of PET data can provide quantitative parameters such as the exchange rate constants of radiolabeled drugs between plasma and tissue compartments, which can be directly related to the function of ABC and SLC transporters localized at blood-tissue interfaces. Whereas considerable knowledge exists with respect to the modeling of PET data in the brain (8–12), less effort has so far been dedicated to the kinetic modeling of PET data in other organs, such as the liver (13). In the case of kinetic modeling of the liver, the dual blood supply to the organ (*i.e.*, by the hepatic artery and the portal vein, PV) is an important factor to be considered (14).

Erlotinib is an epidermal growth factor receptor targeting tyrosine kinase inhibitor used to treat advanced or metastatic non-small cell lung cancer and pancreatic cancer (15). It is mainly excreted via the hepatobiliary route and is a substrate of several ABC and SLC transporters including breast cancer resistance protein (ABCG2), P-glycoprotein (ABCB1), organic anion transporter 3 (SLC22A8), organic cation transporter 2 (SLC22A2), and organic anion-transporting polypeptide 2B1 (SLCO2B1) (16–18). Erlotinib shows large inter-individual differences in its PK, which may be related to variability in membrane transporter function in the liver. We have shown before that PET with [^11^C]erlotinib can be used to study hepatic disposition of erlotinib in humans (18,19). In order to assess the influence of hepatocyte transporters on hepatobiliary excretion of [^11^C]erlotinib, we have previously followed two different approaches. In a first study, we assessed the effect of pre-treatment with an oral therapeutic dose of unlabeled erlotinib on the hepatic disposition of [^11^C]erlotinib (18). In a second study, we assessed the influence of pre-treatment with the prototypical SLCO transporter inhibitor rifampicin on hepatic disposition of [^11^C]erlotinib (19).

In this study, we used these previously published human [^11^C]erlotinib PET data sets (18,19) to evaluate the suitability of different compartment models to describe and quantify the kinetics of [^11^C]erlotinib in the liver and the bile duct/gall bladder. Two different compartment models were assessed (a three-compartment and a four-compartment model) that account for the kinetics of different hepatobiliary sub-structures. We also studied the differences in the PK modeling of [^11^C]erlotinib in the hepatobiliary system with traditional modeling using only a sampled arterial input function (AIF) and when a dual input function (DIF), which takes the 75% contribution of the PV into account, was used as the model input. The ultimate goal of the study was to contribute to finding a generic liver PK model that can be potentially applied to describe the hepatic disposition of other radiolabeled drugs in humans to facilitate future use of PET in the study of membrane transporter function in the liver.

## METHODS

### Data Sets

Clinical data sets were previously published by Bauer *et al.* (18,19). Both clinical studies were registered under EUDRACT number 2015-001593-18 and were approved by the Ethics Committee of the Medical University of Vienna. Written consent was obtained from the study participants before their inclusion into the study.

In both studies, healthy volunteers (*n* = 6 per study) underwent two dynamic [^11^C]erlotinib PET scans of the abdominal region. The time interval between the scans in both studies was approximately 7 days. In the first study, a baseline PET scan was followed by a second PET scan, which was conducted 3 h after oral intake of a 300-mg dose of unlabeled erlotinib. In the second study, a baseline PET scan was followed by a second PET scan, which was conducted following an i.v. infusion of a 600-mg dose of rifampicin, a prototypical SLCO transporter inhibitor, over 30 min. Radioactivity in the upper abdominal region was measured over 90 min. Blood samples were drawn from the radial artery in parallel to the PET imaging. The regions of interest (ROIs) of the liver and extrahepatic bile duct/gall bladder (eBD/GB) were outlined on individual MR to PET co-registered images using PMOD 3.6 (PMOD Technologies, Zurich, Switzerland). The liver ROI was drawn excluding large blood vessels and visible intrahepatic bile ducts. For the delineation of bile duct ROIs early PET images were used whereas the gall bladder was better identifiable on late images. The extracted concentration-time curves (in kBq/mL) were then used for the modeling.

### Liver Dual Input Function

Usually, compartment models developed to analyze PET data include an AIF that describes the radiotracer kinetics in plasma or blood and that is measured by arterial sampling (20). However, the liver receives a dual blood supply (from the hepatic artery and the PV) and, in order to accurately describe the radiotracer concentration presented to the liver, it might be necessary to compute a dual input function (DIF) including both arterial and PV concentrations. Since the PV cannot be sampled in humans, it is assumed that a mathematical method, which has been previously validated in pigs (21), can be used for the computation of the PV input function. In this method, the radiotracer concentration in portal venous blood, *C*_PV_(*t*), is calculated as the convolution integral between the concentration in hepatic arterial blood, *C*_HA_(*t*), and the impulse-response function, *h*(*t*). The blood concentration in the hepatic artery is assumed to be the same as the concentration measured in the radial artery.

1 $$ {C}_{\mathrm{PV}}(t)={\int}_0^th\left(t-\tau \right){C}_{\mathrm{HA}}\left(\tau \right) d\tau $$

The impulse-response function can be characterized by a single parameter *β*, which is radiotracer specific and determines the mean transit time for the passage of radiotracer from the intestinal arteries to the PV (22).

2 $$ h(t)=\frac{\beta }{{\left(\beta +t\right)}^2} $$

The total DIF into the liver can be calculated as

3 $$ {C}_{\mathrm{in}}(t)={f}_{\mathrm{HA}}{C}_{\mathrm{HA}}(t)+{f}_{\mathrm{PV}}{C}_{\mathrm{PV}}(t) $$

where *f*_HA_ (fixed at a value of 0.25) and *f*_PV_ (fixed at a value of 0.75) are the hepatic artery and the PV flow fraction, respectively (23,24).

### Pharmacokinetic Modeling

Individual concentration-time curves for each subject were used to conduct the different compartment analyses using in-house software developed in MATLAB (R2018a, The MathWorks Inc.). Two different compartment models were implemented to analyze [^11^C]erlotinib disposition in the hepatobiliary system. These models assume that the radioactivity signals in the liver and the eBD/GB ROIs measured over the short duration of the PET scan (90 min) only represent unmetabolized [^11^C]erlotinib, which is supported by previous data in mice (25) and rats (26).

#### Four-Compartment Model

**Fig. 1:**
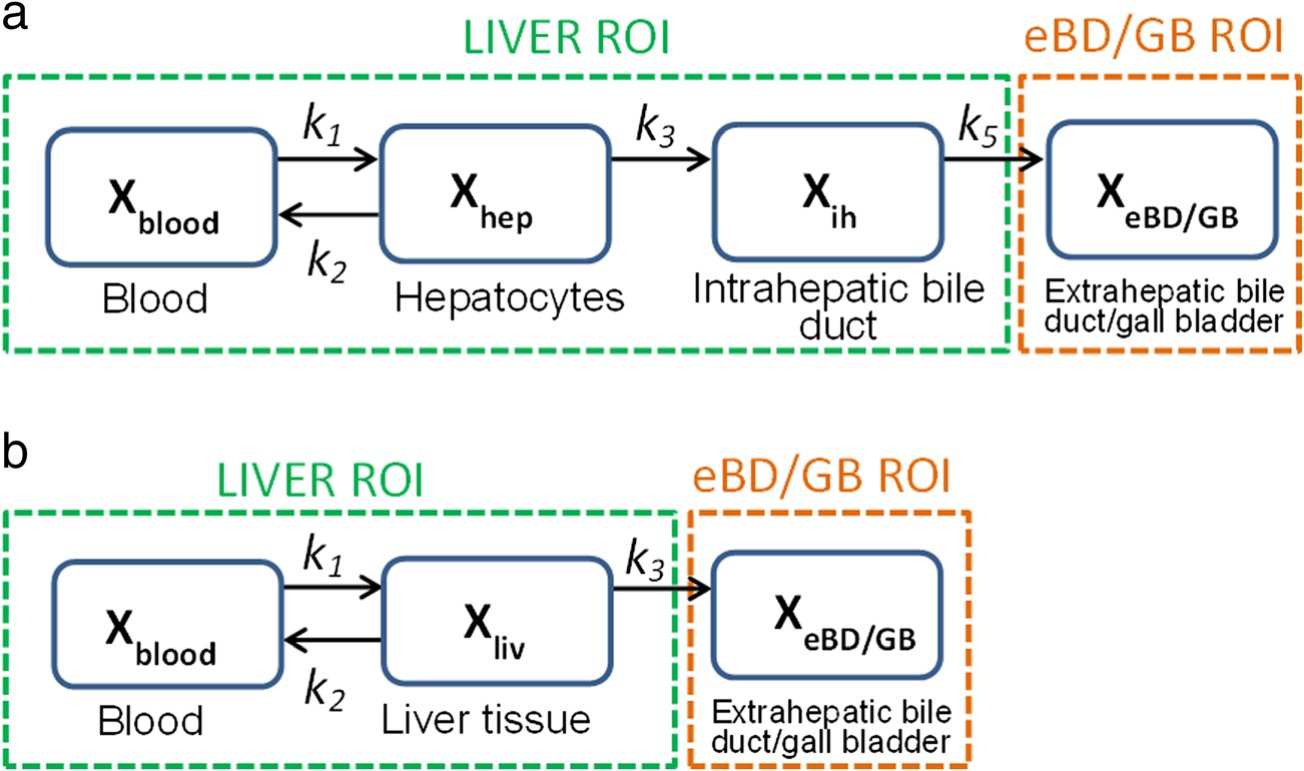
Compartment models for describing hepatic [^11^C]erlotinib disposition. *X*_blood_, *X*_liv_, *X*_hep_, *X*_ih_, and *X*_eBD/GB_ represent the amount of [^11^C]erlotinib in the blood, liver tissue, hepatocyte compartment, intrahepatic bile duct, and compartment representing the extrahepatic bile duct and gall bladder, respectively. The rate constants defining the exchange of [^11^C]erlotinib between blood and liver tissue are defined by *k*_1_ (min^−1^) and *k*_2_ (min^−1^). **a** In the four-compartment model (4C model), *k*_3_ (min^−1^) is the rate constant describing transfer from liver tissue to the intrahepatic bile duct, and *k*_5_ (min^−1^) is the rate constant describing transfer from the intrahepatic bile duct to the extrahepatic bile duct and the gall bladder. This model is divided into two main regions of interest: the liver, composed of the blood, hepatocyte, and intrahepatic bile duct compartments; and the extrahepatic bile duct and gall bladder (eBD/GB), composed of the extrahepatic bile duct and gall bladder compartment. **b** In the three-compartment model (3C model), *k*_3_ (min^−1^) is the rate constant describing transfer from the liver to the extrahepatic bile duct and gall bladder. The 3C model is divided into two main regions of interest: the liver, composed of blood and liver tissue compartments; and eBD/GB, which is composed of the extrahepatic bile duct and gall bladder compartment

The four-compartment (4C) model (Fig. 1a) can be seen as an extension of the PET compartment model proposed by Ørntoft *et al.* (24) to quantify hepatobiliary secretion kinetics of the conjugated radiolabeled bile acid [^11^C]cholylsarcosine. While the model developed in (24) included secretion of the radiotracer out of the PET ROI, the 4C model presented in this work includes an additional compartment that represents the amount of radiotracer in the eBD/GB. The system of ordinary differential equations that describes the transfer of [^11^C]erlotinib between compartments is defined in terms of mass as follows:

4 $$ \frac{d{X}_{\mathrm{hep}}}{dt}={k}_1{X}_{\mathrm{blood}}-\left({k}_2+{k}_3\right){X}_{\mathrm{hep}} $$

5 $$ \frac{d{X}_{\mathrm{ih}}}{dt}={k}_3{X}_{\mathrm{hep}}-{k}_5{X}_{\mathrm{ih}} $$

6 $$ \frac{d{X}_{\mathrm{eBD}/\mathrm{GB}}}{dt}={k}_5{X}_{\mathrm{ih}} $$

where *X*_hep_ and *X*_ih_ are the amount of radiotracer (kBq) in the hepatocytes and intrahepatic bile duct, respectively, and *X*_eBD/GB_ corresponds to the amount of [^11^C]erlotinib in the extrahepatic bile duct and gall bladder region. *k*_1_ (min^−1^) and *k*_2_ (min^−1^) are the rate constants for transfer of [^11^C]erlotinib between blood and liver tissue, *k*_3_ (min^−1^) is the rate constant for transfer of radiotracer from liver into the intrahepatic bile duct, and *k*_5_ (min^−1^) is the rate constant defining the radiotracer transfer from the intrahepatic bile duct to the extrahepatic bile duct/gall bladder. *X*_blood_ is the amount of radiotracer in the blood that can be defined as

7 $$ {X}_{\mathrm{blood}}(t)={V}_{\mathrm{blood}}{C}_{\mathrm{in}}(t) $$

where *V*_blood_ (mL) is the volume of blood in the liver (0.25 mL of blood per mL of liver tissue) (24). *C*_in_(*t*) is the concentration (kBq/mL) of radiotracer in blood used as an input function. Depending on how *C*_in_(t) is defined, two different approaches are presented. The first approach includes an AIF, in which *C*_in_(*t*) is equivalent to the sampled arterial blood concentration (*C*_HA_(*t*)). In the second proposed approach, *C*_in_(*t*) is defined according to Eq. 3*.* The *β* parameter which defines the concentration in the PV (Eqs. 1 and 2) is included as a model parameter that needs to be estimated based on the AIF and the fitted liver and eBD/GB curves.

Since the acquired PET data is in the form of radiotracer concentration in the ROI, the final model concentration must be computed. In this model, we considered two main ROIs: the liver and the eBD/GB. The total amount measured by PET in the ROI, *X*_liver,ROI_(*t*), is the sum of the amounts present in the different sub-regions represented by the compartments (27):

8 $$ {X}_{\mathrm{liver},\mathrm{ROI}}(t)={X}_{\mathrm{blood}}(t)+{X}_{\mathrm{hep}}(t)+{X}_{\mathrm{ih}}(t) $$

Therefore, expressing the amount in terms of concentration, *X*(*t*) = *VC*(*t*), the final concentration in the liver can be calculated as

9 $$ {V}_{\mathrm{liver}}{C}_{\mathrm{liver},\mathrm{ROI}}(t)={V}_{\mathrm{blood}}{C}_{\mathrm{in}}(t)+{X}_{\mathrm{hep}}(t)+{X}_{\mathrm{ih}}(t) $$

10 $$ {C}_{\mathrm{liver},\mathrm{ROI}}(t)=\frac{V_{\mathrm{blood}}}{V_{\mathrm{liver}}}{C}_{\mathrm{in}}(t)+\frac{1}{V_{\mathrm{liver}}}{X}_{\mathrm{hep}}(t)+\frac{1}{V_{\mathrm{liver}}}{X}_{\mathrm{ih}}(t) $$

11 $$ {C}_{\mathrm{liver},\mathrm{ROI}}(t)={V}_{\mathrm{b}}{C}_{\mathrm{in}}(t)+\frac{X_{\mathrm{hep}}(t)+{X}_{\mathrm{ih}}(t)}{V_{\mathrm{liver}}} $$

where *V*_liver_ (mL) is the physiological volume of the liver which was calculated by a previously reported method (28). *V*_b_ is the fraction of blood in the liver, which was set to 0.25 based on literature (24), and *C*_in_(*t*) is either the concentration in the hepatic artery or the DIF depending on the chosen approach.

The final concentration in the eBD/GB can be calculated as follows:

12 $$ {C}_{\mathrm{eBD}/\mathrm{GB},\mathrm{ROI}}(t)=\frac{X_{\mathrm{eBD}/\mathrm{GB}}(t)}{V_{\mathrm{eBD}/\mathrm{GB}}} $$

where *V*_eBD/GB_ is the volume of the extrahepatic bile duct and gall bladder region derived from the PET images. The hepatic uptake clearance (CL_H,uptake_, mL/min/kg of body weight) is calculated by multiplying the blood volume (*V*_blood_) in the liver with the influx rate constant (*k*_1_), and dividing by the weight of the individual (*w*_body_):

13 $$ {\mathrm{CL}}_{\mathrm{H},\mathrm{uptake}}=\frac{k_1{V}_{\mathrm{blood}}}{w_{\mathrm{body}}} $$

The hepatic efflux clearance (CL_H,efflux_, mL/min/kg) can be calculated by multiplying the volume of the intrahepatic bile duct compartment (*V*_ih_, 0.0032 mL per mL of liver tissue) (24) with *k*_5_ and dividing by *w*_body_:

14 $$ {\mathrm{CL}}_{\mathrm{H},\mathrm{efflux}}=\frac{k_5{V}_{\mathrm{ih}}}{w_{\mathrm{body}}} $$

#### Three-Compartment Model

The three-compartment (3C) model (Fig. 1b) is a simplification of the 4C model in which the liver tissue is represented as a single compartment instead of being divided into hepatocytes and intrahepatic bile duct. This model was previously proposed to represent the distribution of [^11^C]erlotinib in the liver and eBD/GB in a similar approach (18), but instead of including an input function into the model, the concentration of radiotracer in the blood was solved for in the model as well as for the liver and eBD/GB data. The 3C model presented in this work is described by the following system of ordinary differential equations

15 $$ \frac{d{X}_{\mathrm{liv}}}{dt}={k}_1{X}_{\mathrm{blood}}-\left({k}_2+{k}_3\right){X}_{\mathrm{liv}} $$

16 $$ \frac{d{X}_{\mathrm{eBD}/\mathrm{GB}}}{dt}={k}_3{X}_{\mathrm{liv}} $$

where *X*_liv_ is the amount of radiotracer (kBq) in the liver tissue and *X*_eBD/GB_ corresponds to the amount of radiotracer in the extrahepatic bile duct and gall bladder. *k*_1_ (min^−1^) and *k*_2_ (min^−1^) are the rate constants defining the transfer of radiotracer between blood and liver tissue, and *k*_3_ (min^−1^) is the rate constant that defines the transfer from the liver to the extrahepatic bile duct and gall bladder. *X*_blood_(*t*) (the amount of [^11^C]erlotinib in the blood) is defined by Eq. 5*.* The definition of *C*_in_(*t*) will also determine whether the input function is simply an AIF or a DIF including both PV and hepatic artery concentrations (Eqs. 1–3).

The total amount in the liver ROI is given as

17 $$ {X}_{\mathrm{liv}\mathrm{er},\mathrm{ROI}}(t)={X}_{\mathrm{blood}}(t)+{X}_{\mathrm{liv}}(t) $$

Therefore, representing the amount in terms of concentration

18 $$ {V}_{\mathrm{liv}\mathrm{er}}{C}_{\mathrm{liv}\mathrm{er},\mathrm{ROI}}(t)={V}_{\mathrm{blood}}{C}_{\mathrm{in}}(t)+{X}_{\mathrm{liv}}(t) $$

19 $$ {C}_{\mathrm{liv}\mathrm{er},\mathrm{ROI}}(t)={V}_{\mathrm{b}}{C}_{\mathrm{in}}(t)+\frac{X_{\mathrm{liv}}(t)}{V_{\mathrm{liv}\mathrm{er}}} $$

where *V*_liver_ (mL) is the physiological volume of the liver, *V*_b_ is the fraction of blood in the liver (set to 0.25), and *C*_in_(*t*) is either the concentration in the hepatic artery or the DIF depending on the chosen approach. The total concentration in the eBD/GB ROI is defined in Eq. 12. CL_H,uptake_ (mL/min/kg) is calculated as described in Eq. 13. CL_H,efflux_ (mL/min/kg) can be calculated by multiplying the volume of liver tissue (*V*_liv_) with *k*_3_ and dividing by the weight of the subject (*w*_body_):

20 $$ C{L}_{\mathrm{H},\mathrm{efflux}}=\frac{k_3{V}_{\mathrm{liv}}}{w_{\mathrm{body}}} $$

The kinetic parameters were estimated by weighted nonlinear least squares using MATLAB (*lsqnonlin* function). The *β* parameter was also included as a model parameter which was estimated based on the fits as well as on the DIF as defined in Eqs. 1–3. The goodness-of-fit was evaluated by visual inspection and Akaike’s Information Criterion (AIC) (29). The model with the smallest AIC was considered to be the most suitable to represent the radiotracer concentration-time profiles in the different tissues of interest:

21 $$ \mathrm{AIC}=N\ln \left(\mathrm{WRSS}\right)+2P $$

where *N* is the number of data points, *P* is the number of estimated parameters in the model and WRSS is the weighted residual sum of squares evaluated at the minimum:

22 $$ \mathrm{WRSS}={\sum}_{j=1}^N{w}_j{\left[{C}_{\mathrm{obs}}\left({t}_j\right)-{C}_{\mathrm{model}}\left({t}_j\right)\right]}^2 $$

where *t*_*j*_ is the time point, *C*_obs_(*t*_*j*_) is the concentration observed in the PET scan *versus* time, and *C*_model_ is the concentration obtained with the model. *w*_*j*_ is the weight of the *j*th data point defined as

23 $$ {w}_j=\frac{\varDelta {t}_j}{C_{\mathrm{obs}}\left({t}_j\right)\ {e}^{-\lambda {t}_j}} $$

where ∆*t*_*j*_ is the length of the PET measuring intervals, and *λ* is the decay constant defined by the half-life (*t*_1/2_) of the radionuclide (*i.e.*, 20.4 min for ^11^C) (30,31):

24 $$ \lambda =\frac{\ln (2)}{t_{1/2}} $$

### Statistical Analysis

Statistically significant differences in pharmacokinetic parameters between studies were assessed using two-tailed paired or unpaired *t* tests. Differences in the AIC values among models were analyzed by ordinary one-way ANOVA test. Pearson correlation coefficient *r* was calculated to assess correlations. Statistical analyses were performed in Prism 7.0 software (GraphPad Software, La Jolla, CA, USA). The level of statistical significance was set to a *p* value of less than 0.05. All values are given as mean ± standard deviation (SD).

## RESULTS

### Analysis of [^11^C]Erlotinib + Erlotinib Study

Data from the six study participants who underwent two different PET scans was analyzed by the two different models developed by us. The first baseline scan consisted of an i.v. bolus injection of [^11^C]erlotinib, while the second scan included an oral administration of erlotinib (300 mg) at 3 h prior to the radiotracer injection (18).

**Fig. 2:**
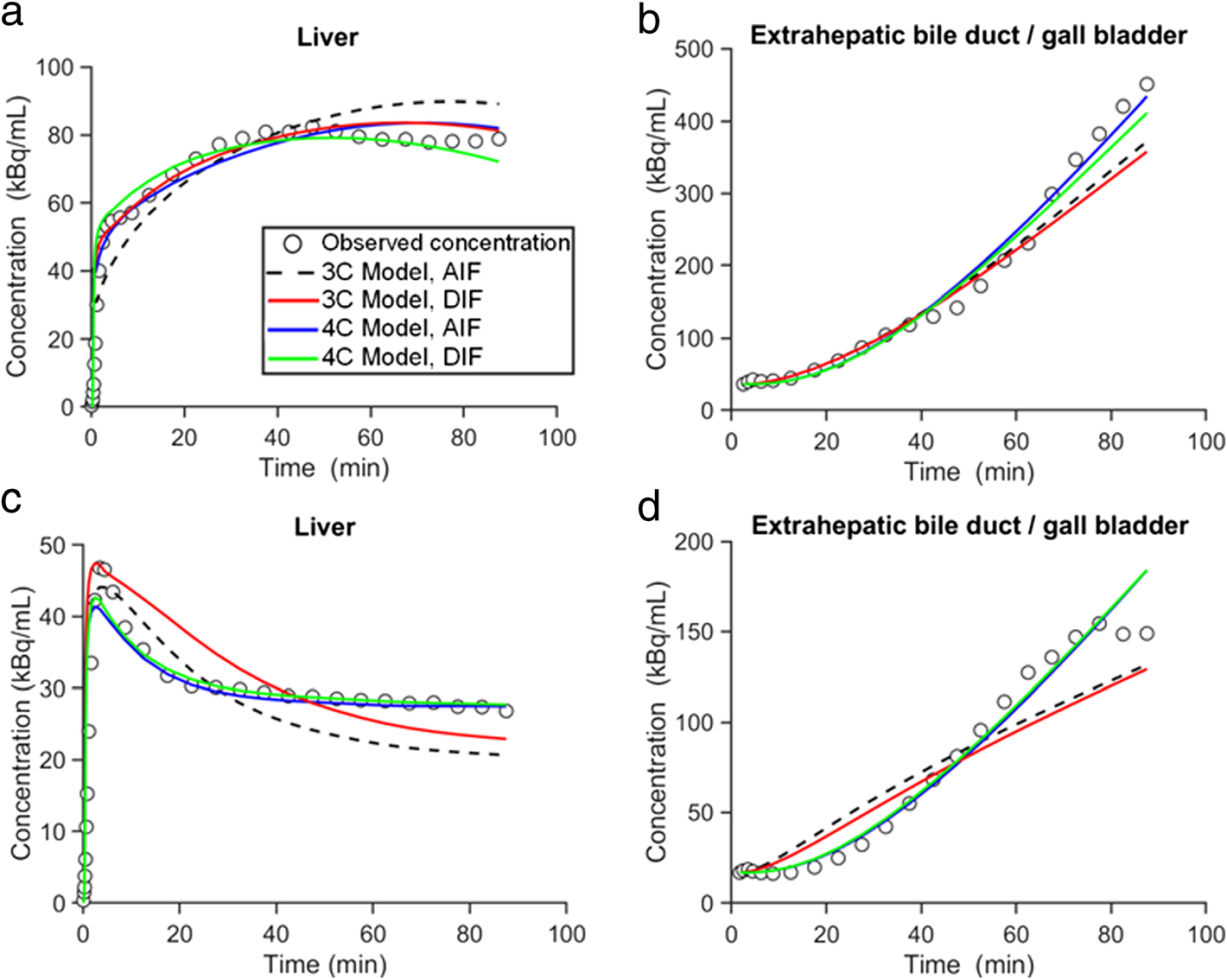
Concentration-time profiles of observed data and model predictions in one individual (p12) for the [^11^C]erlotinib + erlotinib data set. Concentration-time profiles in the liver and extrahepatic bile duct/gall bladder in the baseline scan (**a**, **b**) and in the scan acquired after oral pre-treatment with unlabeled erlotinib (**c**, **d**). Observed data is represented with open black circles; modeled data is represented with colored lines (AIF, arterial input function; DIF, dual input function; 3C, three compartment; 4C, four compartment)

The model comparisons for each ROI in both scans (for the subject in whom the best fits were obtained) are depicted in Fig. 2. The model comparisons in the other five subjects are shown in the Supplemental Material (Supplemental Figs. 1–5). The goodness-of-fit was evaluated by visual inspection and AIC values. Visually, all models represented the radiotracer disposition in liver and eBD/GB over time reasonably well. However, the 4C model, which included a second liver tissue compartment representing the intrahepatic bile duct, improved the fits for the liver compartment in the second scan (Fig. 2c), and therefore, it more precisely represented the decrease in radiotracer concentration in the liver when unlabeled erlotinib was administered before the radiotracer. Although the 4C model provided slightly better fits for the eBD/GB region, fits did not considerably differ between models. Supplemental Table I shows the AIC values of the 3C and 4C models with either AIF or DIF, which vary among individuals. The AIC values did not show statistically significant differences in the performance of the different implemented models. However, slightly lower AIC values (indicating better goodness-of-fit) were observed for the models using the DIF. While the 3C model appeared to perform better in the baseline scan (mean AIC value, 3C model 249 ± 41; 4C model 262 ± 38), the 4C model provided lower AIC values for scan 2 (mean AIC value, 3C model 223 ± 42, 4C model 212 ± 38).

**Fig. 3:**
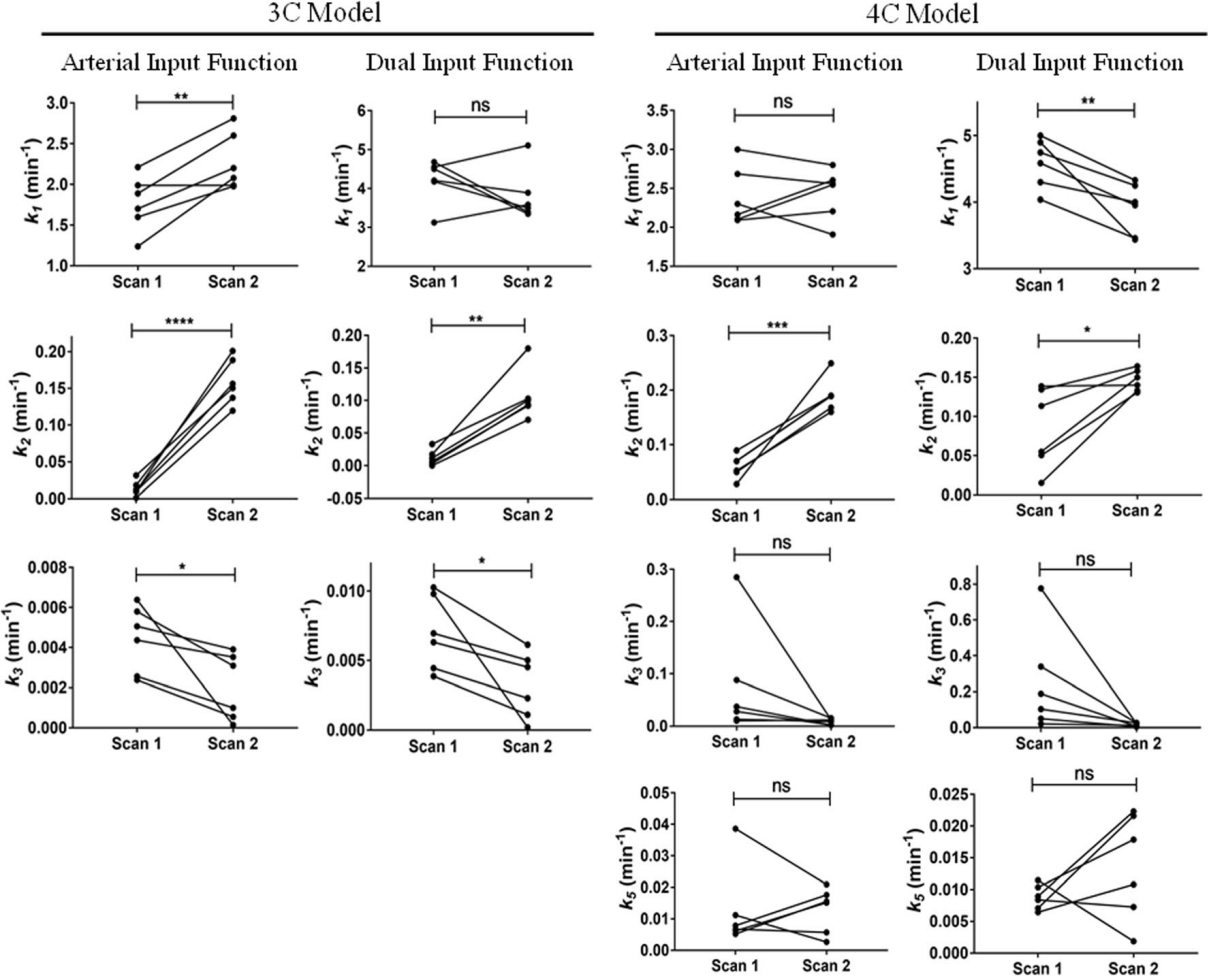
Changes in outcome parameters between the first and second scans for the [^11^C]erlotinib + erlotinib data set. *k*_1_, *k*_2_, *k*_3_, and *k*_5_ values obtained in individual subjects for each model in scan 1 and scan 2. ns, not significant; *p ≤ 0.05; **p ≤ 0.01; ***p ≤ 0.005; ****p ≤ 0.0001, two-tailed paired *t* test

Parameter estimates for the 3C models (using AIF and DIF) are shown in Table I while parameter estimates for the 4C models are shown in Table II. Numerical identifiability was satisfactory in all studies. Parameter precision (represented as percent coefficient of variation, %CV) was poor only for *β*, especially in the 4C model and in subjects p12 and p13 in the 3C model. Changes in model outcome parameters from scan 1 to scan 2 in all models are depicted in Fig. 3. The *k*_1_ estimates were significantly higher for the DIF than for the AIF approach in both the 3C and the 4C models. However, this parameter did not follow any consistent trend between scan 1 and scan 2 among models. The value of *k*_1_ was significantly increased between scans in the 3C model using AIF, while it was significantly reduced from scan 1 to scan 2 in the 4C model using DIF. The other two implemented models did not show any significant variation for the *k*_1_ value between scans. Furthermore, *k*_2_ was significantly increased from the baseline scan to the second scan in all described models. Even though the value of *k*_3_ decreased from scan 1 to scan 2 in all models, this change was only significant in the 3C model. *k*_5_ was not significantly altered between scans in the 4C models. Hepatic uptake and efflux clearances (CL_H,uptake_ and CL_H,efflux_) were also calculated and values are given in Supplemental Table II. The changes in CL_H,uptake_ from scan 1 to scan 2 were comparable to those for *k*_1_ (Supplemental Fig. 6). The CL_H,efflux_ in the 3C model was significantly reduced from scan 1 to scan 2, while for the 4C model, there was no significant alteration of CL_H,efflux_ between scans (Supplemental Fig. 7).

**Table I:** Parameter Estimates of the 3C Model for the [^11^C]Erlotinib + Erlotinib Data Set

Subject	Arterial input function^*a*^			Dual input function^*b*^			
	*k*_1_ (min^−1^)	*k*_2_ (min^−1^)	*k*_3_ (min^−1^)	*k*_1_ (min^−1^)	*k*_2_ (min^−1^)	*k*_3_ (min^−1^)	*β* (min)
Baseline scan							
p12	1.700 (2)	0.001 (28)	0.004 (1)	4.207 (2)	0.0001 (71)	0.007 (1)	0.899 (152)
p13	1.989 (4)	0.032 (5)	0.003 (2)	4.501 (7)	0.033 (7)	0.004 (2)	0.812 (272)
p14	1.599 (2)	0.012 (4)	0.005 (1)	4.178 (3)	0.011 (10)	0.010 (1)	0.801 (42)
p15	2.212 (2)	0.010 (5)	0.006 (1)	4.683 (4)	0.003 (13)	0.006 (1)	1.720 (57)
p24	1.888 (3)	0.018 (4)	0.002 (1)	4.553 (5)	0.017 (4)	0.004 (1)	1.753 (74)
p30	1.238 (2)	0.010 (4)	0.006 (1)	3.124 (3)	0.006 (6)	0.010 (1)	1.714 (22)
Mean ± SD	1.771 ± 0.339	0.016 ± 0.009	0.004 ± 0.002	4.208 ± 0.567	0.012 ± 0.012	0.007 ± 0.003	1.283 ± 0.490
Second scan (after oral erlotinib)							
p12	2.201 (7)	0.119 (7)	0.004 (2)	3.891 (7)	0.070 (6)	0.005 (2)	0.805 (81)
p13	2.299 (14)	0.150 (14)	0.001 (3)	3.349 (15)	0.103 (15)	0.002 (3)	1.649 (58)
p14	1.978 (6)	0.156 (6)	0.004 (1)	3.499 (4)	0.100 (4)	0.006 (1)	0.799 (52)
p15	2.810 (11)	0.201 (10)	0.003 (2)	3.394 (6)	0.094 (6)	0.005 (2)	0.899 (47)
p24	2.600 (11)	0.188 (10)	0.001 (3)	5.110 (10)	0.180 (9)	0.001 (3)	1.558 (49)
p30	2.080 (11)	0.137 (10)	0.0001 (3)	3.585 (7)	0.092 (6)	0.0002 (2)	2.154 (53)
Mean ± SD	2.328 ± 0.319	0.159 ± 0.031	0.002 ± 0.002	3.805 ± 0.668	0.107 ± 0.038	0.003 ± 0.002	1.311 ± 0.561

Values in parentheses represent percent coefficient of variation (%CV) of the parameter (parameter precision), calculated as (SE(*p*_*i*_)/*p*_*i*_)100, where *p*_*i*_ is the parameter estimate and SE is the asymptotic standard error of the mean of *p*_*i*_. *k*_1_ and *k*_2_ are the exchange rate constants between blood and liver tissue, and *k*_3_ is the rate constant describing transfer from the liver to the extrahepatic bile duct and gall bladder. *β* is the mean transit time for the passage of radiotracer from intestinal arteries to the portal vein

^*a*^3C model using the blood sampled from the radial artery as the model input function. *β* estimation is not necessary since portal vein concentration is not calculated

^*b*^3C model using the dual input (combination of hepatic artery and portal vein) as the model input function

**Table II:** Parameter Estimates of the 4C Model for the [^11^C]Erlotinib + Erlotinib Data Set

Subject	Arterial input function^*a*^				Dual input function^*b*^				
	*k*_1_ (min^−1^)	*k*_2_ (min^−1^)	*k*_3_ (min^−1^)	*k*_5_ (min^−1^)	*k*_1_ (min^−1^)	*k*_2_ (min^−1^)	*k*_3_ (min^−1^)	*k*_5_ (min^−1^)	*β* (min)
Baseline scan									
p12	3.000 (7)	0.052 (16)	0.088 (7)	0.006 (1)	4.748 (6)	0.015 (45)	0.103 (8)	0.010 (1)	0.827 (139)
p13	2.300 (8)	0.050 (10)	0.013 (10)	0.007 (7)	4.899 (13)	0.050 (20)	0.021 (13)	0.008 (8)	0.799 (172)
p14	2.102 (5)	0.028 (8)	0.010 (4)	0.039 (9)	4.038 (11)	0.134 (36)	0.339 (15)	0.009 (1)	2.152 (58)
p15	2.092 (13)	0.089 (41)	0.285 (21)	0.008 (2)	4.587 (21)	0.113 (148)	0.777 (43)	0.007 (1)	1.899 (36)
p24	2.686 (8)	0.069 (13)	0.028 (9)	0.005 (4)	5.000 (23)	0.055 (44)	0.050 (11)	0.007 (3)	1.807 (158)
p30	2.164 (5)	0.070 (7)	0.037 (8)	0.011 (3)	4.301 (17)	0.139 (32)	0.189 (16)	0.012 (2)	1.482 (164)
Mean ± SD	2.391 ± 0.372	0.060 ± 0.021	0.077 ± 0.106	0.013 ± 0.013	4.596 ± 0.368	0.084 ± 0.051	0.247 ± 0.284	0.009 ± 0.002	1.494 ± 0.570
Second scan (after oral erlotinib)									
p12	2.801 (9)	0.160 (12)	0.015 (9)	0.015 (7)	4.250 (12)	0.133 (15)	0.026 (7)	0.018 (4)	2.169 (55)
p13	1.907 (16)	0.168 (19)	0.009 (16)	0.006 (12)	3.439 (16)	0.131 (21)	0.012 (18)	0.007 (13)	0.787 (173)
p14	2.547 (9)	0.249 (10)	0.012 (5)	0.021 (5)	3.457 (12)	0.164 (12)	0.027 (5)	0.022 (3)	1.899 (45)
p15	2.205 (11)	0.190 (14)	0.012 (8)	0.018 (7)	3.956 (17)	0.158 (14)	0.022 (10)	0.022 (8)	1.889 (75)
p24	2.560 (13)	0.189 (13)	0.002 (17)	0.016 (21)	4.330 (18)	0.150 (16)	0.005 (12)	0.011 (12)	0.800 (85)
p30	2.606 (12)	0.189 (12)	0.002 (19)	0.003 (18)	4.000 (19)	0.140 (17)	0.006 (10)	0.002 (8)	1.050 (88)
Mean ± SD	2.438 ± 0.323	0.191 ± 0.031	0.009 ± 0.006	0.013 ± 0.007	3.905 ± 0.382	0.146 ± 0.014	0.016 ± 0.010	0.014 ± 0.008	1.432 ± 0.621

Values in parentheses represent the precision of the parameter estimates (%CV). *k*_1_ and *k*_2_ are the exchange rate constants between blood and liver tissue, *k*_3_ is the transfer rate constant from liver tissue to the intrahepatic bile duct, and *k*_5_ is the transfer rate constant from the intrahepatic bile duct to the extrahepatic bile duct and gall bladder. *β* is the mean transit time for the passage of radiotracer from intestinal arteries to the portal vein

^*a*^4C model using the blood sampled from the radial artery as the model input function. *β* estimation is not necessary since portal vein concentration is not calculated

^*b*^4C model using the dual input (combination of hepatic artery and portal vein) as the model input function

### Analysis of [^11^C]Erlotinib + Rifampicin Study

**Fig. 4:**
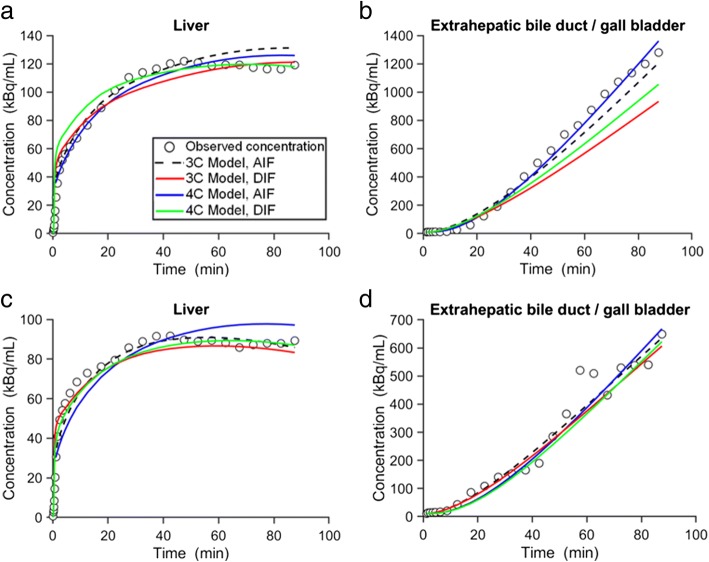
Concentration-time profiles of observed data and model predictions in one individual (p40) for the [^11^C]erlotinib + rifampicin data set. Concentration-time profiles in the liver and extrahepatic bile duct/gall bladder in the baseline scan (**a**, **b**) and in the scan acquired after i.v. administration of rifampicin (**c**, **d**). Observed data is represented with open black circles; modeled data is represented with colored lines (AIF, arterial input function; DIF, dual input function; 3C, three compartment; 4C, four compartment)

Six study participants underwent two [^11^C]erlotinib PET scans: a baseline scan and a second scan after i.v. infusion of rifampicin (19). Model comparisons for both ROIs in the baseline scan and the scan after rifampicin infusion (for the subject with the best fits) are depicted in Fig. 4. The model comparisons in the other five subjects are shown in the Supplemental Material (Supplemental Figs. 8–12). Visually, all models showed a good agreement between the observed and the fitted concentrations. The 4C model provided slightly better fits, especially for eBD/GB. The AIC values did not show significant differences between different models (Supplemental Table III). However, lowest AIC values, indicating best fits, were found for the 3C model using the AIF and the 4C model using the DIF in the first scan (mean AIC value for 3C/AIF model 272 ± 49, 3C/DIF model 315 ± 91, 4C/AIF model 283 ± 13, and 4C/DIF model 271 ± 36) and for the 3C model using AIF in the second scan (mean AIC value for 3C/AIF model 255 ± 28, 3C/DIF model 288 ± 38, 4C/AIF model 269 ± 25, and 4C/DIF model 265 ± 41).

**Fig. 5:**
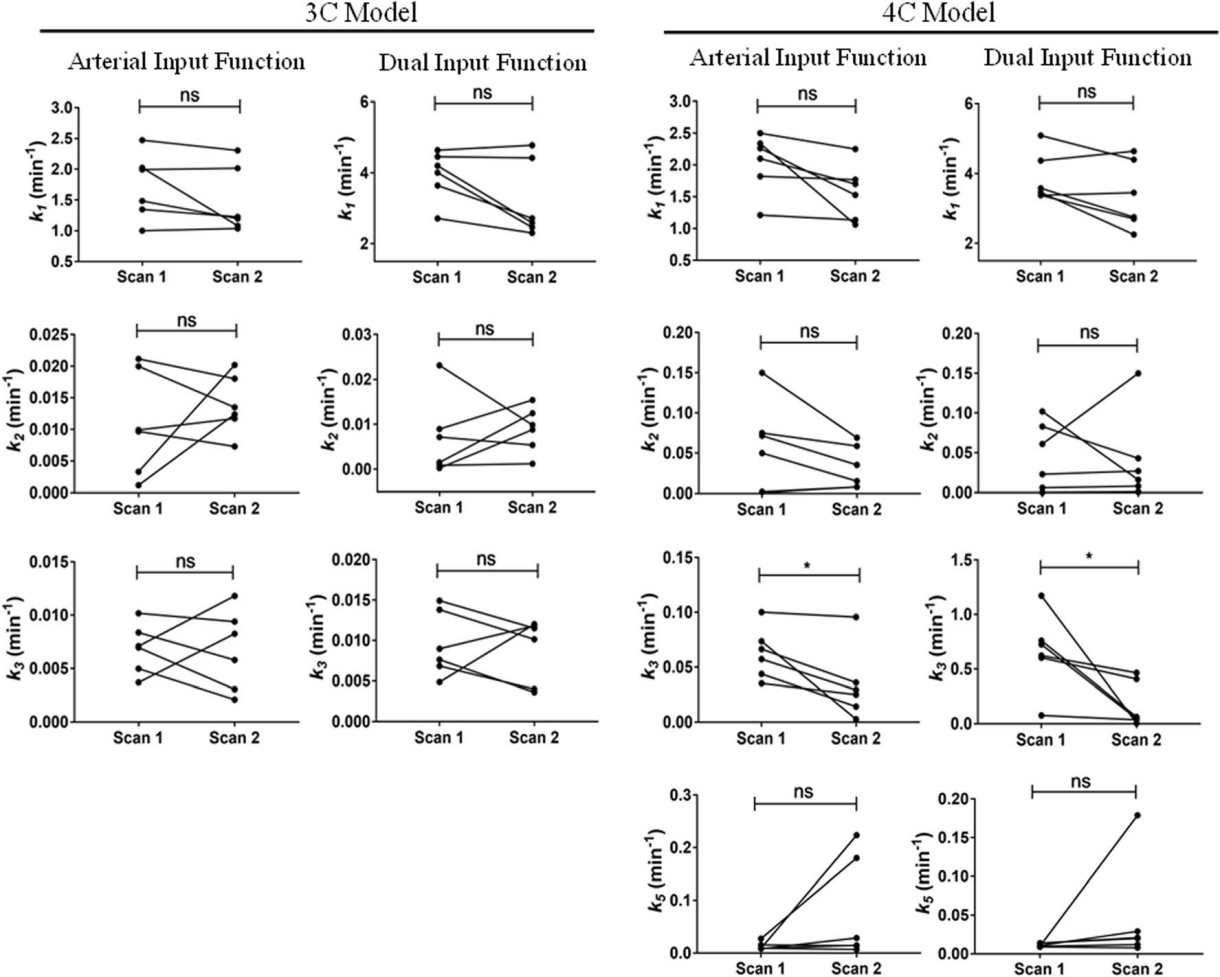
Changes in outcome parameters between the first and second scans for the [^11^C]erlotinib + rifampicin data set. *k*_1_, *k*_2_, *k*_3_, and *k*_5_ values obtained in individual subjects for each model in scan 1 and scan 2. ns, not significant; *p ≤ 0.05, two-tailed paired *t* test

Parameter estimates for the 3C models using both AIF and DIF are shown in Table III and parameter estimates from the 4C models are shown in Table IV. Parameter precision (%CV) was poorer for *k*_2_ in the DIF model in some of the individuals for both the 3C and the 4C models. Changes in outcome parameters between scans are depicted in Fig. 5. The value of *k*_1_ showed a tendency for a moderate decrease from scan 1 to scan 2 in most individuals for all the implemented models, but statistical significance was not reached. No model showed significant changes for the *k*_2_ value from scan 1 to scan 2. The value of *k*_3_ was reduced from scan 1 to scan 2 in almost all subjects for all models. This reduction was, however, only significant in the 4C model with both AIF and DIF. *k*_5_ in the 4C model was not significantly altered between scan 1 and scan 2. The values for CL_H,uptake_ and CL_H,efflux_ are shown in Supplemental Table IV. There was a trend towards a decrease in CL_H,uptake_ from scan 1 to scan 2 in all models (Supplemental Fig. 13), similar to the reduction in *k*_1_, but none of these changes were statistically significant. No significant changes were found for CL_H,efflux_ in any of the implemented models (Supplemental Fig. 14).

**Table III:** Parameter Estimates of the 3C Model for the [^11^C]Erlotinib + Rifampicin Data Set

Subject	Arterial input function			Dual input function			
	*k*_1_ (min^−1^)	*k*_2_ (min^−1^)	*k*_3_ (min^−1^)	*k*_1_ (min^−1^)	*k*_2_ (min^−1^)	*k*_3_ (min^−1^)	*β* (min)
Baseline scan							
p37	1.000 (3)	0.021 (4)	0.004 (1)	2.711 (8)	0.023 (10)	0.005 (1)	2.170 (69)
p38	2.024 (2)	0.020 (2)	0.005 (1)	4.202 (3)	0.009 (10)	0.008 (1)	0.608 (49)
p39	1.346 (1)	0.003 (10)	0.008 (1)	3.643 (5)	0.002 (30)	0.014 (1)	0.802 (40)
p40	1.994 (1)	0.001 (20)	0.007 (1)	4.456 (5)	0.0002(277)	0.007 (2)	1.342 (84)
p41	2.471 (1)	0.010 (3)	0.007 (1)	4.639 (5)	0.001 (101)	0.009 (1)	0.758 (44)
p42	1.485 (4)	0.010 (13)	0.010 (3)	4.008 (7)	0.007 (28)	0.015 (5)	0.800 (138)
Mean ± SD	1.720 ± 0.538	0.011 ± 0.008	0.007 ± 0.002	3.943 ± 0.697	0.007 ± 0.009	0.010 ± 0.004	1.080 ± 0.590
Second scan (after i.v. rifampicin)							
p37	1.037 (2)	0.018 (4)	0.008 (1)	2.304 (3)	0.010 (9)	0.012 (1)	2.122 (41)
p38	1.082 (2)	0.014 (4)	0.002 (1)	2.589 (3)	0.015 (5)	0.004 (1)	0.823 (74)
p39	1.227 (3)	0.020 (4)	0.006 (1)	2.715 (3)	0.013 (9)	0.010 (2)	0.758 (56)
p40	2.016 (1)	0.012 (3)	0.003 (1)	4.422 (1)	0.009 (5)	0.004 (1)	0.797 (28)
p41	2.305 (1)	0.007 (6)	0.011 (1)	4.781 (3)	0.001 (76)	0.012 (1)	0.601 (69)
p42	1.197 (2)	0.012 (6)	0.009 (1)	2.462 (5)	0.005 (24)	0.012 (2)	0.727 (73)
Mean ± SD	1.477 ± 0.542	0.014 ± 0.005	0.007 ± 0.004	3.212 ± 1.091	0.009 ± 0.005	0.009 ± 0.004	0.971 ± 0.569

For legend to this table, see Table I legend

**Table IV:** Parameter Estimates of the 4C Model for the [^11^C]Erlotinib + Rifampicin Data Set

Subject	Arterial input function			Dual input function					
	*k*_1_ (min^−1^)	*k*_2_ (min^−1^)	*k*_3_ (min^−1^)	*k*_5_ (min^−1^)	*k*_1_ (min^−1^)	*k*_2_ (min^−1^)	*k*_3_ (min^−1^)	*k*_5_ (min^−1^)	*β* (min)
Baseline scan									
p37	2.100 (11)	0.150 (14)	0.035 (10)	0.008 (4)	3.463 (12)	0.102 (18)	0.076 (12)	0.011 (2)	0.879 (59)
p38	2.339 (7)	0.072 (9)	0.057 (9)	0.009 (2)	3.579 (6)	0.083 (57)	0.724 (21)	0.009 (1)	1.755 (54)
p39	2.264 (7)	0.075 (8)	0.066 (10)	0.016 (3)	3.378 (1)	0.023 (16)	0.759 (16)	0.014 (1)	1.157 (25)
p40	1.821 (2)	0.002 (78)	0.074 (6)	0.009 (1)	5.091 (2)	0.006 (69)	1.172 (29)	0.010 (2)	0.839 (60)
p41	2.501 (6)	0.050 (18)	0.100 (7)	0.009 (1)	4.371 (3)	0.0003(21)	0.622 (18)	0.010 (1)	1.708 (90)
p42	1.211 (7)	0.001(342)	0.044 (17)	0.027 (10)	3.376 (18)	0.061(184)	0.603 (37)	0.014 (2)	1.678 (60)
Mean ± SD	2.039 ± 0.467	0.058 ± 0.056	0.063 ± 0.023	0.013 ± 0.008	3.876 ± 0.704	0.046 ± 0.042	0.659 ± 0.353	0.011 ± 0.002	1.336 ± 0.429
Second scan (after i.v. rifampicin)									
p37	1.700 (4)	0.069 (7)	0.025 (7)	0.029 (7)	2.250 (26)	0.016 (61)	0.036 (26)	0.029 (23)	2.17 (106)
p38	1.062 (5)	0.035 (6)	0.029 (8)	0.007 (4)	2.752 (8)	0.043 (17)	0.046 (7)	0.008 (2)	1.626 (82)
p39	1.527 (6)	0.059 (11)	0.036 (9)	0.014 (4)	2.700 (4)	0.027 (24)	0.062 (17)	0.020 (7)	1.614 (92)
p40	1.770 (2)	0.008 (6)	0.003 (1)	0.224 (9)	4.399 (3)	0.008 (7)	0.004 (1)	0.179 (7)	1.993 (75)
p41	2.251 (5)	0.015 (35)	0.096 (7)	0.015 (2)	4.641 (4)	0.001 (27)	0.464 (20)	0.012 (1)	2.168 (93)
p42	1.135 (3)	0.008 (14)	0.014 (2)	0.180 (14)	3.452 (18)	0.150 (52)	0.408 (25)	0.021 (2)	2.167(143)
Mean ± SD	1.574 ± 0.440	0.032 ± 0.027	0.034 ± 0.033	0.078 ± 0.097	3.366 ± 0.976	0.041 ± 0.056	0.170 ± 0.208	0.045 ± 0.066	1.956 ± 0.269

For legend to this table, see Table II legend

## DISCUSSION

To advance the applicability of PET imaging as an upcoming tool in the study of hepatic transporter function, we evaluated different compartment modeling approaches for the analysis of previously acquired human PET data with [^11^C]erlotinib (18,19), which we considered as a small-molecule drug model, which undergoes transporter-mediated hepatobiliary excretion.

Traditional PET PK models are based on physiologically based pharmacokinetic models, in which the compartments correspond to predefined organs or tissues interconnected by blood flow, lymph flow, or other biochemical fluxes (32). Typically, the input of the system in PET kinetic modeling is a directly measured arterial blood or plasma curve (radioactivity in the blood/plasma as a function of time) termed AIF (11). Even though the blood is a physical compartment, it is typically not a mathematical compartment in PET kinetic models since it is a directly measured curve rather than being solved for (11).

The liver receives blood from the hepatic artery (~ 25%) from which it acquires oxygenated blood, and from the PV (~ 75%) that provides deoxygenated blood containing newly absorbed nutrients, drugs, and, occasionally, toxins from the gastrointestinal tract (33). Thus, considering only AIF as a source of radioactivity to a liver PK model might not be accurate. Since direct blood sampling from the PV is not feasible in humans, several previous modeling approaches have applied image-derived PV blood curves (34–36). However, this approach is prone to imaging artifacts such as partial volume effects or respiratory motion, which are challenging to correct for and which may affect the accuracy of the model-derived parameter estimates (37). Therefore, we evaluated in our study an approach which relied on a mathematically derived DIF obtained from the sampled AIF (21,24,38). The estimated DIF was computed as a combination of the AIF and the PV contribution to the liver blood supply. The mathematical model used to obtain the PV concentration as a function of time was previously validated in pigs for other radiotracers (22). The *β* parameter that allowed the computation of the concentration in the PV (Eqs. 1 and 2) is radiotracer specific and can be experimentally determined if blood samples from the PV and from an artery are obtained. A high *β* value means loss of radiotracer in the intestine which may be due to transporter-mediated active secretion into the intestinal lumen, resulting in a low PV input function as compared to the AIF (Supplemental Fig. 15). On the other hand, a low *β* value would mean no or little loss of radiotracer in the intestinal lumen and comparable peaks of the PV and the AIF (22).

In previous studies with the radiolabeled conjugated bile acid [^11^C]cholylsarcosine, the *β* value was derived from studies in pigs, in which the PV blood curve was directly sampled, and then applied to the analysis of the human data, under the assumption that the *β* value does not differ between humans and pigs for this radiotracer (21,24). As for [^11^C]erlotinib no *β* value was available from experiments in pigs, we introduced *β* as a model parameter to be estimated, similar to the approach by Winterdahl *et al.* (22). However, our results showed high inter-individual variability of the *β* parameter as well as a large %CV (Tables I, II, III, and IV) indicating that its estimation might not be accurate. Our *β* estimates ranged from 0.6 to 2.2 min which was in comparable range to previously described radiotracers in pigs ([^11^C]methylglucose, 2-[^18^F]fluoro-2-deoxy-d-galactose, [^15^O]water), which showed some degree of loss upon intestinal passage (22). In fact, in the case of [^11^C]erlotinib, active intestinal secretion may be mediated by ABCG2 and ABCB1 located in the luminal membrane of the intestinal epithelial cells. These assumptions are supported by previous data published by Bauer *et al.* (18), which showed a reduction in the intestinal concentration of radioactivity in the PET scan performed after oral pre-treatment with unlabeled erlotinib presumably due to partial saturation of ABCG2/ABCB1-mediated intestinal secretion of [^11^C]erlotinib. However, as our *β* estimates were not very reliable, no firm conclusions can be drawn regarding changes in *β* values between scans. Interestingly, the fitting results (see Figs. 2 and 4) did not present any substantial improvements for the implementation of the DIF as compared with the use of only the AIF. Nevertheless, slightly lower AIC values were obtained when implementing the mathematically derived DIF for most of the individuals, which indicated that the DIF represented more accurately the true liver input function. The fact that the PV input function has a considerably lower peak than the AIF (Supplemental Fig. 15) resulted in an increase of *k*_1_ estimates from the AIF to the DIF approach (Tables I, II, III, and IV). However, as we were not able to directly obtain the PV input function in humans to experimentally validate the mathematically derived DIF, using the AIF as the model input may serve as a valid alternative to represent the hepatic disposition of [^11^C]erlotinib. In this way, uncertainties in the model results arising from the *β* parameter can be avoided. However, depending on the characteristics of the radiotracer, inclusion of the DIF might be essential in order to obtain accurate model fits. Therefore, future efforts should be directed towards improved measurement of image-derived PV input functions in humans.

In addition to the model input function, kinetic models for PET typically involve one or two compartments that describe the kinetics of the radiotracer in the tissue of interest (11). The one-tissue compartment model (two-compartment) describes the bidirectional flux of radiotracer between blood and tissue, while the two-tissue compartment model (three-compartment) includes a second tissue compartment that represents additional binding and/or metabolism of the radiotracer inside the tissue. In our study, we assumed that no metabolism of [^11^C]erlotinib occurred over the short duration of the PET scan, so that the model represented the exchange of unmetabolized radiotracer between different compartments. This assumption was supported by previous data in humans showing that the majority of radioactivity in plasma (> 95% at all studied time points) was found to be composed of unmetabolized [^11^C]erlotinib both for baseline PET scans and for PET scans acquired after administration of unlabeled erlotinib or rifampicin (18,19). Moreover, in mice and rats, chromatographic analysis revealed that the majority of radioactivity in liver tissue homogenate (approximately 60% in mice at 25 min after radiotracer injection and approximately 96% in rats at 15 min after radiotracer injection) represented unchanged [^11^C]erlotinib (25,26). The models developed in this work included an additional compartment that did represent not only the state of the radiotracer in the liver but also its secretion into the extrahepatic bile duct and gall bladder. Two models were employed in this study: a 4C model (three tissue compartments, Fig. 1a) and a 3C model (two tissue compartments, Fig. 1b). In the 4C model, the intrahepatic bile duct and the eBD/GB were considered as distinct compartments with rate constants describing the transfer of radiotracer from the liver into the intrahepatic bile duct (*k*_3_) and from the latter into the eBD/GB (*k*_5_). The radiotracer PK in eBD/GB was directly measured in the PET scans and therefore included into the model analysis, while the intrahepatic bile ducts represented a compartment that was not directly visible in the PET scans. The transporter-mediated secretion of [^11^C]erlotinib into bile as mediated by canalicular hepatocyte ABC transporters should therefore be primarily reflected by the *k*_3_ parameter. On the other hand, in the 3C model, the liver compartment and the intrahepatic bile duct compartment were lumped together, so that the *k*_3_ parameter mainly reflected the transfer of radiotracer from the hepatocytes into the eBD/GB.

The [^11^C]erlotinib + erlotinib PET data analyzed in this study have already been kinetically evaluated utilizing another 3C model, which did not provide satisfactory fits of the liver and eBD/GB data (18). In contrast to this, our results showed that both employed models could appropriately represent the radiotracer kinetics in the liver and in the eBD/GB. However, the 4C model better represented the decrease in liver distribution of radiotracer in scan 2, in which [^11^C]erlotinib was administered after oral pre-treatment with unlabeled erlotinib. Previously conducted *in vitro* transport studies demonstrated that SLCO2B1 is a high-affinity, low-capacity transporter of [^11^C]erlotinib (18). This led to the suggestion that hepatic SLCO2B1 contributed, next to passive diffusion, to uptake of [^11^C]erlotinib from the blood into the liver as reflected by a decrease in the liver-to-blood ratio of [^11^C]erlotinib when an oral therapeutic dose of unlabeled erlotinib was given before the PET scan (18). Counterintuitively, the previous kinetic analysis had shown that the decrease in liver concentrations observed in scan 2 was reflected by an increase in *k*_2_, rather than by a decrease in *k*_1_, as would be expected from saturation of a hepatic uptake transporter. The lack of a *k*_1_ reduction in scan 2 has been explained in a way that initial liver uptake of [^11^C]erlotinib was mainly mediated by passive diffusion with only a small contribution by SLCO2B1. It has been hypothesized that the increase in *k*_2_ was caused by saturation of SLCO2B1 activity, which decreased the ability of [^11^C]erlotinib to re-enter from the blood into the liver after the initial uptake phase (18). Importantly, all compartment models evaluated in this study corroborated the *k*_2_ increase in scan 2 obtained in the previous analysis (18) (Fig. 3). Moreover, the two employed 3C models revealed a significant reduction of *k*_3_ in scan 2 (Fig. 3). This decrease in *k*_3_ was also significant in the 4C DIF model when the baseline scan values from both studies (erlotinib and rifampicin) were grouped in a single baseline scan and compared to the values from the scan with the co-administration of erlotinib (Supplemental Fig. 16). Such a *k*_3_ reduction was not obtained in the previous analysis (18), but would be expected based on previously published preclinical PET data in mice (25). In the mouse study, co-injection of [^11^C]erlotinib with unlabeled erlotinib (10 mg/kg) led to a pronounced reduction in CL_H,efflux_ of [^11^C]erlotinib, determined with integration plot analysis, presumably due to saturation of ABCG2/ABCB1-mediated transport of [^11^C]erlotinib from the liver into the bile (25).

For the second study, in which [^11^C]erlotinib PET scans were performed after administration of the prototypical SLCO transporter inhibitor rifampicin, there were no significant changes in the uptake or backflux of the radiotracer between the blood and the liver (Fig. 5). This contrasted with a previous analysis based on integration plot analysis and kinetic modeling using a liver model implemented in freely available *iFit* software (www.liver.dk/ifit.html), which revealed a moderate reduction of *k*_1_ in the scan recorded after rifampicin infusion (19). This moderate effect of rifampicin on *k*_1_ was attributed to rifampicin exerting only a weak inhibitory effect on SLCO2B1 at the attained plasma concentrations, as opposed to its strong inhibitory effect on SLCO1B1 and SLCO1B3, for which [^11^C]erlotinib is not a substrate (18,19). The *k*_1_ reduction was further supported by data in mice, which received a higher dose of rifampicin than for the human study leading to a pronounced decrease in *k*_1_ (19). Our present data also showed a trend for *k*_1_ decreases, but statistical significance was not reached, most likely due to the small sample size of the study (Fig. 5). However, when *k*_1_ values from the baseline scan in both studies (erlotinib and rifampicin) were grouped as a single baseline scan, the reduction in *k*_1_ when rifampicin was co-administered became significant in all the implemented models except in the 3C AIF (Supplemental Fig. 16). In accordance with the previous analysis (19), we found in the employed 4C models a significant reduction in *k*_3_, which was most likely caused by inhibition of ABCG2/ABCB1-mediated secretion of [^11^C]erlotinib from the liver into the bile by rifampicin.

Hepatic uptake and efflux clearances were also calculated for every implemented model (Supplemental Tables II and IV). Changes in CL_H,uptake_ were similar to the *k*_1_ changes in both studies. While there were no significant changes in CL_H,uptake_ when unlabeled erlotinib was administered before the second scan, there was a trend for a decrease in CL_H,uptake_ when rifampicin was administered before the second scan, which was consistent with a weak inhibitory effect of rifampicin on SLCO2B1-mediated hepatic uptake of [^11^C]erlotinib (19). The CL_H,uptake_ values, when the DIF was implemented, were of the same order of magnitude as the liver blood flow (21 mL/min/kg body weight) (23). However, the CL_H,uptake_ values for the AIF model were considerably higher than those of the hepatic artery blood flow (4.3 mL/min/kg body weight) but much lower than those of the total liver blood flow. This indicated that the mathematical approximation of the DIF using the model-derived PV input function and the sampled AIF might be more accurate to represent the uptake mechanisms of [^11^C]erlotinib than only the use of the AIF. The value of CL_H,efflux_ between the 3C model and the 4C model cannot be directly compared since the clearance is calculated from different rate constants (*k*_3_ in 3C model and *k*_5_ in 4C model) and from different physiological volumes (liver tissue volume and intrahepatic bile duct volume for the 3C and 4C models, respectively) corresponding to the defined compartments. This caused a significant reduction in CL_H,efflux_ from the 3C to the 4C model since the volume in the intrahepatic bile duct (*V*_ih_) was defined as 3.2 mL of bile per liter of liver tissue (24).

Both data sets have been previously analyzed using a graphical analysis method termed integration plot analysis (18,19). This approach allows determining the clearances or the rate constants for initial uptake of radioactivity from the blood into the liver and for efflux of radioactivity from the liver into the bile and has been used in several other studies to assess the impact of liver transporters on the liver kinetics of different PET tracers (39,40). We found a significant positive correlation between the kinetic parameters obtained in this study (using AIF) with the respective values from integration plot analysis (Supplemental Fig. 17). However, integration plot analysis is based on arterial blood, so that *k*_uptake,liver_ values do not represent the transfer rate constant from sinusoidal blood into the liver. Moreover, the rate constant describing the backflux of radioactivity from the liver into the blood (*k*_2_) cannot be determined using integration plot analysis. For certain drugs, this parameter may be influenced by the activity of efflux transporters located in the basolateral membrane of hepatocytes (*e.g.*, multidrug resistance-associated proteins 3 and 4, ABCC3 and ABCC4) and is therefore of major relevance to be determined. Moreover, the estimation of *k*_efflux,liver_ is not straightforward in some occasions using integration plot analysis since a linear phase of the plot cannot be always identified leading to inconclusive results.

An alternative approach to analyze the present data may be the implementation of a population-based PK model, which may facilitate the statistical comparison of the individual models, enable a better comparison of the parameter estimates using a covariate approach, and finally improve the identifiability of the *β* parameter. Even though population-based PK models usually involve large numbers of heterogeneous patients in which few samples are taken per individual so that it is not possible to characterize the individual’s PK, such models have also been previously applied to PET data (41–43). However, as population-based PK modeling requires dedicated software packages, which are not available to us, this analysis was not pursued in the present study.

As shown in this study, compartment analysis of PET data can be used to assess transporter-mediated DDIs in the liver. It can be anticipated that our analysis approach may also be applicable to assess pathophysiological changes or polymorphisms in the transporter-encoding genes as sources of PK variability. Our models may also find application to assess hepatic disposition of other radiolabeled drugs or to obtain improved readouts for diagnostic liver PET or SPECT tracers.

## CONCLUSION

We developed 3C and 4C models to describe the kinetics of [^11^C]erlotinib in the hepatobiliary system. Both the models provided acceptable fits of the observed data, but the 4C model provided a richer kinetic picture, including an additional compartment that represented the radiotracer disposition in the intrahepatic bile duct that was not visible in the PET scan but also accounted for the total liver radioactivity. Changes in model outcome parameters between scans were consistent with the involvement of basolateral hepatocyte uptake and canalicular efflux transporters in the hepatobiliary clearance of [^11^C]erlotinib. Contrary to our expectations, our results demonstrated that inclusion of a DIF did not lead to substantial improvements in model fits. However, the benefit of a DIF may depend on the radiotracer being employed, so that future efforts should be directed towards defining improved algorithms for partial volume and motion correction of image-derived PV input functions in humans. The models developed in this work represent a step forward in applying PET as a tool to assess the impact of hepatic transporters on drug disposition and their involvement in DDIs.

## Electronic Supplementary Material

ESM 1 (DOCX 9.38 mb)

## Abbreviations

3C: Three-compartment model
4C: Four-compartment model
ABC: ATP-binding cassette
AIC: Akaike’s information criterion
AIF: Arterial input function
DDI: Drug-drug interaction
DIF: Dual input function
eBD/GB: Extrahepatic bile duct and gall bladder
PET: Positron emission tomography
PK: Pharmacokinetics
PV: Portal vein
ROI: Region of interest
SLC: Solute carrier

## References

[1] Kusuhara H, Sugiyama Y (2009). In vitro-in vivo extrapolation of transporter-mediated clearance in the liver and kidney. Drug Metab Pharmacokinet.

[2] Giacomini K, Huang S, Tweedie D (2010). Membrane transporters in drug development. Nat Rev Drug Discov.

[3] Langer O (2016). Use of PET imaging to evaluate transporter-mediated drug-drug interactions. J Clin Pharmacol.

[4] Patilea-Vrana G, Unadkat JD (2016). Transport vs. metabolism: what determines the pharmacokinetics and pharmacodynamics of drugs? Insights from the extended clearance model. Clin Pharmacol Ther.

[5] Chu X, Korzekwa K, Elsby R, Fenner K, Galetin A, Lai Y, Matsson P, Moss A, Nagar S, Rosania GR, Bai JPF, Polli JW, Sugiyama Y, Brouwer KLR (2013). Intracellular drug concentrations and transporters: measurement, modeling, and implications for the liver. Clin Pharmacol Ther.

[6] Tournier N, Stieger B, Langer O (2018). Imaging techniques to study drug transporter function in vivo. Pharmacol Ther.

[7] Pastor CM, Langer O, Van Beers BE. Liver imaging and hepatobiliary contrast media. Contrast Media Mol Imaging. 2018;2487405. 10.1155/2018/2487405 PMC6151230 30271310

[8] Gunn RN, Gunn SR, Turkheimer FE, Aston JA, Cunningham VJ (2002). Positron emission tomography compartmental models: a basis pursuit strategy for kinetic modeling. J Cereb Blood Flow Metab.

[9] Innis RB, Cunningham VJ, Delforge J, Fujita M, Gjedde A, Gunn RN, Holden J, Houle S, Huang SC, Ichise M, Iida H, Ito H, Kimura Y, Koeppe RA, Knudsen GM, Knuuti J, Lammertsma AA, Laruelle M, Logan J, Maguire RP, Mintun MA, Morris ED, Parsey R, Price JC, Slifstein M, Sossi V, Suhara T, Votaw JR, Wong DF, Carson RE (2007). Consensus nomenclature for in vivo imaging of reversibly binding radioligands. J Cereb Blood Flow Metab.

[10] Mintun MA, Raichle ME, Kilbourn MR, Wooten GF, Welch MJ (1984). A quantitative model for the in vivo assessment of drug binding sites with positron emission tomography. Ann Neurol.

[11] MORRIS E, ENDRES C, SCHMIDT K, CHRISTIAN B, MUZICJR R, FISHER R (2004). Kinetic Modeling in Positron Emission Tomography. Emission Tomography.

[12] Cunningham VJ, Lammertsma AA (1995). Radioligand studies in brain: kinetic analysis of PET data. Med Chem Res.

[13] Keiding S, Sørensen M, Frisch K, Gormsen LC, Munk OL (2018). Quantitative PET of liver functions. Am J Nucl Med Mol Imaging.

[14] Cui Y, Bai J (2005). Comparison of parameter estimations using dual-input and arterial-input in liver kinetic studies of FDG metabolism. IEEE Eng Med Biol.

[15] Hammerman PS, Janne PA, Johnson BE (2009). Resistance to epidermal growth factor receptor tyrosine kinase inhibitors in non-small cell lung cancer. Clin Cancer Res.

[16] Elmeliegy MA, Carcaboso AM, Tagen M, Bai F, Stewart CF (2011). Role of ATP-binding cassette and solute carrier transporters in erlotinib CNS penetration and intracellular accumulation. Clin Cancer Res.

[17] Kodaira H, Kusuhara H, Ushiki J, Fuse E, Sugiyama Y (2010). Kinetic analysis of the cooperation of P-glycoprotein (P-gp/Abcb1) and breast cancer resistance protein (Bcrp/Abcg2) in limiting the brain and testis penetration of erlotinib, flavopiridol, and mitoxantrone. J Pharmacol Exp Ther.

[18] Bauer M, Matsuda A, Wulkersdorfer B, Philippe C, Traxl A, Özvegy-Laczka C, Stanek J, Nics L, Klebermass EM, Poschner S, Jäger W, Patik I, Bakos É, Szakács G, Wadsak W, Hacker M, Zeitlinger M, Langer O (2018). Influence of OATPs on hepatic disposition of erlotinib measured with positron emission tomography. Clin Pharmacol Ther.

[19] Bauer M, Traxl A, Matsuda A, Karch R, Philippe C, Nics L, Klebermass EM, Wulkersdorfer B, Weber M, Poschner S, Tournier N, Jäger W, Wadsak W, Hacker M, Wanek T, Zeitlinger M, Langer O (2018). Effect of rifampicin on the distribution of [^11^C]erlotinib to the liver, a translational PET study in humans and in mice. Mol Pharm.

[20] Kamasak ME (2012). Computation of variance in compartment model parameter estimates from dynamic PET data. Med Phys.

[21] Sørensen M, Munk OL, Ørntoft NW, Frisch K, Andersen KJ, Mortensen FV (2016). Hepatobiliary secretion kinetics of conjugated bile acids measured in pigs by [^11^C]cholylsarcosine PET. J Nucl Med.

[22] Winterdahl M, Keiding S, Sørensen M, Mortensen FV, Alstrup AK, Munk OL (2011). Tracer input for kinetic modelling of liver physiology determined without sampling portal venous blood in pigs. Eur J Nucl Med Mol I.

[23] Davies B, Morris T (1993). Physiological parameters in laboratory animals and humans. Pharm Res.

[24] Ørntoft NW, Munk OL, Frisch K, Ott P, Keiding S, Sørensen M (2017). Hepatobiliary transport kinetics of the conjugated bile acid tracer [^11^C]CSar quantified in healthy humans and patients by positron emission tomography. J Hepatol.

[25] Traxl A, Wanek T, Mairinger S, Stanek J, Filip T, Sauberer M, Muller M, Kuntner C, Langer O (2015). Breast cancer resistance protein and p-glycoprotein influence in vivo disposition of [^11^C]erlotinib. J Nucl Med.

[26] Amor D, Goutal S, Marie S, Caillé F, Bauer M, Langer O, Auvity S, Tournier N (2018). Impact of rifampicin-inhibitable transport on the liver distribution and tissue kinetics of erlotinib assessed with PET imaging in rats. EJNMMI Res.

[27] Bertoldo A, Peltoniemi P, Oikonen V, Knuut J, Nuutila P, Cobelli C (2001). Kinetic modeling of [^18^F]FDG in skeletal muscle by PET: a four-compartment five-rate-constant model. Am J Physiol Endocrinol Metab.

[28] Vauthey JN, Abdalla EK, Doherty DA, Gertsch P, Fenstermacher MJ, Loyer EM (2002). Body surface area and body weight predict total liver volume in Western adults. Liver Transpl.

[29] Yamaoka K, Nakagawa T, Uno T (1977). Application of Akaike’s Information Criterion (AIC) in the evaluation of linear pharmacokinetic equations. J Pharmacokinet Biopharm.

[30] Mazoyer BM, Huesman RH, Budinger TF, Knittel BL (1986). Dynamic PET data analysis. J Comput Assist Tomogr.

[31] Lammertsma AA, Dittrich S, van den Hoff J, Maguire RP (2010). Receptor kinetics - modelling and practical approach. PET pharmacokinetic course manual 2010.

[32] Jones HM, Gardner IB, Watson KJ (2009). Modelling and PBPK simulation in drug discovery. AAPS J.

[33] Tortora GJ, Derrickson B. The digestive system. In: Guarascio M, Rama L, Myers L, editors. Principles of anatomy and physiology. 15th edn. Hoboken, NJ: John Wiley & Sons; 2014. p. 898–952.

[34] Gormsen LC, Søndergaard E, Christensen NL, Jakobsen S, Nielsen EHT, Munk OL, Tolbod LP, Jessen N, Nielsen S (2018). Metformin does not affect postabsorptive hepatic free fatty acid uptake, oxidation or resecretion in humans: a 3-month placebo controlled clinical trial in patients with type 2 diabetes and healthy controls. Diabetes Obes Metab.

[35] Kudomi N, Jarvisalo MJ, Kiss J, Borra R, Viljanen A, Viljanen T (2009). Non-invasive estimation of hepatic glucose uptake from [^18^F]FDG PET images using tissue-derived input functions. Eur J Nucl Med Mol Imaging.

[36] Chouhan MD, Bainbridge A, Atkinson D, Punwani S, Mookerjee RP, Lythgoe MF, Taylor SA (2017). Improved hepatic arterial fraction estimation using cardiac output correction of arterial input functions for liver DCE MRI. Phys Med Biol.

[37] Zanotti-Fregonara P, Chen K, Liow JS, Fujita M, Innis RB (2011). Image-derived input function for brain PET studies: many challenges and few opportunities. J Cereb Blood Flow Metab.

[38] Munk OL, Bass L, Roelsgaard K, Bender D, Hansen SB, Keiding S (2001). Liver kinetics of glucose analogs measured in pigs by PET: importance of dual-input blood sampling. J Nucl Med.

[39] Kaneko K, Tanaka M, Ishii A, Katayama Y, Nakaoka T, Irie S, Kawahata H, Yamanaga T, Wada Y, Miyake T, Toshimoto K, Maeda K, Cui Y, Enomoto M, Kawamura E, Kawada N, Kawabe J, Shiomi S, Kusuhara H, Sugiyama Y, Watanabe Y (2018). A clinical quantitative evaluation of hepatobiliary transport of [^11^C]dehydropravastatin in humans using positron emission tomography. Drug Metab Dispos.

[40] Takashima T, Kitamura S, Wada Y, Tanaka M, Shigihara Y, Ishii H, Ijuin R, Shiomi S, Nakae T, Watanabe Y, Cui Y, Doi H, Suzuki M, Maeda K, Kusuhara H, Sugiyama Y, Watanabe Y (2012). PET imaging-based evaluation of hepatobiliary transport in humans with (15R)-^11^C-TIC-Me. J Nucl Med.

[41] Kissel J, Port RE, Zaers J, Bellemann ME, Strauss LG, Haberkorn U, Brix G (1999). Noninvasive determination of the arterial input function of an anticancer drug from dynamic PET scans using the population approach. Med Phys.

[42] Li J, Kim S, Shields AF, Douglas KA, McHugh CI, Lawhorn-Crews JM (2016). Integrating dynamic positron emission tomography and conventional pharmacokinetic studies to delineate plasma and tumor pharmacokinetics of FAU, a prodrug bioactivated by thymidylate synthase. J Clin Pharmacol.

[43] Gandia P, Jaudet C, Everaert H, Heemskerk J, Vanbinst AM, de Mey J, Duerinck J, Neyns B, de Ridder M, Chatelut E, Concordet D (2017). Population pharmacokinetic approach applied to positron emission tomography: computed tomography for tumor tissue identification in patients with glioma. Clin Pharmacokin.

