# Supplementary material for: Towards Improved Pharmacokinetic Models for the Analysis of Transporter-Mediated Hepatic Disposition of Drug Molecules with Positron Emission Tomography
Source: AAPS J. 2019 Apr 29;21(4):61. doi: 10.1208/s12248-019-0323-0 (PMC6488550; doi:10.1208/s12248-019-0323-0)
Supplement: Supplementary file 1 — (DOCX 9.38 mb) [file 12248_2019_323_MOESM1_ESM.docx]

**SupplementaL material**

**Towards improved pharmacokinetic models for the analysis of transporter-mediated hepatic disposition of drug molecules with positron emission tomography**

Irene Hernández Lozano, Rudolf Karch, Martin Bauer, Matthias Blaickner, Akihiro Matsuda, Beatrix Wulkersdorfer, Marcus Hacker, Markus Zeitlinger, and Oliver Langer

**Supplemental Table I.** AIC values of the 3C and 4C models for the [^11^C]erlotinib + erlotinib data set.

|  | AIC Values | | | |
| --- | --- | --- | --- | --- |
|  | 3C Model | | 4C Model | |
| Subject | AIF | DIF | AIF | DIF |
|  | *Baseline Scan* | | | |
| p12 | 228 | 221 | 241 | 228 |
| p13 | 277 | 284 | 295 | 303 |
| p14 | 253 | 246 | 273 | 238 |
| p15 | 276 | 311 | 286 | 317 |
| p24 | 252 | 232 | 274 | 245 |
| p30 | 228 | 201 | 264 | 243 |
|  | *Second Scan (after oral erlotinib)* | | | |
| p12 | 258 | 234 | 240 | 199 |
| p13 | 254 | 218 | 265 | 262 |
| p14 | 207 | 194 | 214 | 151 |
| p15 | 275 | 298 | 254 | 214 |
| p24 | 262 | 220 | 252 | 237 |
| p30 | 230 | 175 | 237 | 207 |

AIF, arterial input function

DIF, dual input function

3C, three-compartment model

4C, four-compartment model

**Supplemental Table II.** Uptake (*CL_H,uptake_*) and efflux (*CL_H,efflux_*) clearance values for all implemented models for the [^11^C]erlotinib + erlotinib data set.

|  | 3C Model | | | | | | | 4C Model | | | | | | | |  |
| --- | --- | --- | --- | --- | --- | --- | --- | --- | --- | --- | --- | --- | --- | --- | --- | --- |
|  | AIF | | | DIF | | | | AIF | | | | DIF | | | |  |
| Subject | *CL_H,uptake_* (mL/min/kg) | | *CL_H,efflux_* (mL/min/kg) | | *CL_H,uptake_* (mL/min/kg) | | *CL_H,efflux_* (mL/min/kg) | | *CL_H,uptake_* (mL/min/kg) | | *CL_H,efflux_* (mL/min/kg) | | *CL_H,uptake_* (mL/min/kg) | | *CL_H,efflux_* (mL/min(kg) | |
|  | *Baseline Scan* | | | | | | | | | | | | | | |  |
| p12 | 9.598 | 0.067 | | 23.707 | | 0.104 | | 16.939 | | 0.001 | | 26.817 | | 0.001 | |  |
| p13 | 10.556 | 0.037 | | 23.878 | | 0.063 | | 12.200 | | 0.001 | | 25.998 | | 0.001 | |  |
| p14 | 8.778 | 0.074 | | 22.875 | | 0.151 | | 11.528 | | 0.003 | | 21.944 | | 0.001 | |  |
| p15 | 12.112 | 0.086 | | 25.316 | | 0.093 | | 11.552 | | 0.001 | | 25.201 | | 0.001 | |  |
| p24 | 9.371 | 0.032 | | 22.672 | | 0.052 | | 12.950 | | 0.0003 | | 24.909 | | 0.0004 | |  |
| p30 | 6.300 | 0.090 | | 16.375 | | 0.138 | | 11.338 | | 0.001 | | 22.575 | | 0.001 | |  |
|  | *Second Scan (after oral erlotinib)* | | | | | | | | | | | | | | |  |
| p12 | 12.415 | 0.053 | | 12.437 | | 0.076 | | 15.805 | | 0.001 | | 24.000 | | 0.001 | |  |
| p13 | 12.200 | 0.014 | | 17.778 | | 0.033 | | 10.080 | | 0.0004 | | 18.039 | | 0.001 | |  |
| p14 | 10.861 | 0.057 | | 19.194 | | 0.090 | | 13.722 | | 0.002 | | 19.208 | | 0.002 | |  |
| p15 | 15.402 | 0.046 | | 18.650 | | 0.066 | | 12.112 | | 0.001 | | 21.739 | | 0.002 | |  |
| p24 | 12.950 | 0.008 | | 25.417 | | 0.013 | | 12.950 | | 0.001 | | 21.415 | | 0.001 | |  |
| p30 | 11.025 | 0.002 | | 18.900 | | 0.003 | | 13.650 | | 0.0002 | | 21.000 | | 0.0001 | |  |

AIF, arterial input function

DIF, dual input function

3C, three-compartment model

4C, four-compartment model

**Supplemental Table III.** Akaike’s Information Criterion (AIC) values of the 3C and 4C models for the [^11^C]erlotinib + rifampicin data set.

|  | AIC Values | | | |
| --- | --- | --- | --- | --- |
|  | 3C Model | | 4C Model | |
| Subject | AIF | DIF | AIF | DIF |
|  | *Baseline Scan* | | | |
| p37 | 275 | 298 | 289 | 297 |
| p38 | 241 | 250 | 263 | 225 |
| p39 | 265 | 266 | 279 | 250 |
| p40 | 276 | 335 | 292 | 288 |
| p41 | 214 | 252 | 274 | 245 |
| p42 | 360 | 489 | 300 | 320 |
|  | *Second Scan (after i.v. rifampicin)* | | | |
| p37 | 268 | 333 | 268 | 305 |
| p38 | 256 | 257 | 247 | 233 |
| p39 | 238 | 235 | 246 | 205 |
| p40 | 223 | 276 | 275 | 257 |
| p41 | 244 | 303 | 260 | 285 |
| p42 | 302 | 321 | 315 | 306 |

AIF, arterial input function

DIF, dual input function

3C, three-compartment model

4C, four-compartment model

**Supplemental Table IV.** Uptake (*CL_H,uptake_*) and efflux (*CL_H,efflux_*) clearance values for all implemented models for the [^11^C]erlotinib + rifampicin data set.

|  | 3C Model | | | | | | | 4C Model | | | | | | | |
| --- | --- | --- | --- | --- | --- | --- | --- | --- | --- | --- | --- | --- | --- | --- | --- |
|  | AIF | | | DIF | | | | AIF | | | | DIF | | | |
| Subject | *CL_H,uptake_* (mL/min/kg) | | *CL_H,efflux_* (mL/min/kg) | | *CL_H,uptake_* (mL/min/kg) | | *CL_H,efflux_* (mL/min/kg) | | *CL_H,uptake_* (mL/min/kg) | | *CL_H,efflux_* (mL/min/kg) | | *CL_H,uptake_* (mL/min/kg) | | *CL_H,efflux_* (mL/min/kg) |
|  | *Baseline Scan* | | | | | | | | | | | | | | |
| p37 | 4.958 | 0.043 | | 13.395 | | 0.065 | | 10.420 | | 0.0005 | | 17.160 | | 0.001 | |
| p38 | 11.400 | 0.077 | | 23.938 | | 0.116 | | 13.338 | | 0.001 | | 20.413 | | 0.001 | |
| p39 | 7.798 | 0.131 | | 20.984 | | 0.216 | | 13.172 | | 0.001 | | 19.703 | | 0.0005 | |
| p40 | 10.914 | 0.103 | | 24.471 | | 0.100 | | 9.986 | | 0.0007 | | 27.929 | | 0.001 | |
| p41 | 13.867 | 0.107 | | 26.000 | | 0.136 | | 14.044 | | 0.0007 | | 24.533 | | 0.0007 | |
| p42 | 8.507 | 0.155 | | 22.685 | | 0.227 | | 6.863 | | 0.002 | | 19.164 | | 0.001 | |
|  | *Second Scan (after i.v. rifampicin)* | | | | | | | | | | | | | | |
| p37 | 5.160 | 0.110 | | 11.411 | | 0.160 | | 8.437 | | 0.002 | | 11.160 | | 0.002 | |
| p38 | 6.163 | 0.031 | | 14.763 | | 0.055 | | 6.038 | | 0.0005 | | 15.675 | | 0.001 | |
| p39 | 7.406 | 0.091 | | 15.859 | | 0.158 | | 8.922 | | 0.001 | | 15.734 | | 0.002 | |
| p40 | 11.086 | 0.046 | | 24.243 | | 0.059 | | 9.871 | | 0.016 | | 24.143 | | 0.013 | |
| p41 | 12.918 | 0.166 | | 26.844 | | 0.178 | | 12.673 | | 0.001 | | 26.044 | | 0.001 | |
| p42 | 6.753 | 0.143 | | 13.945 | | 0.175 | | 6.466 | | 0.013 | | 19.562 | | 0.002 | |

AIF, arterial input function

DIF, dual input function

3C, three-compartment model

4C, four-compartment model

c


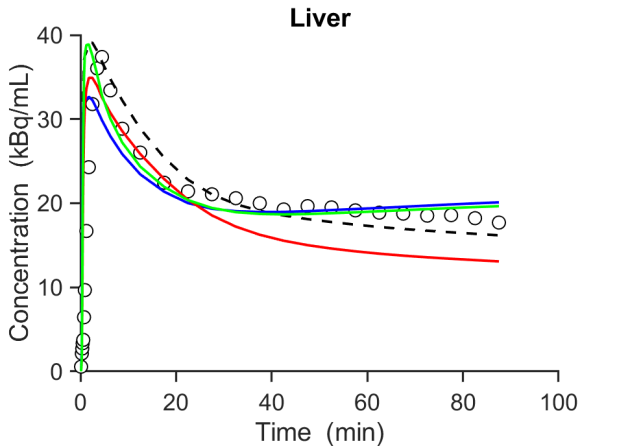

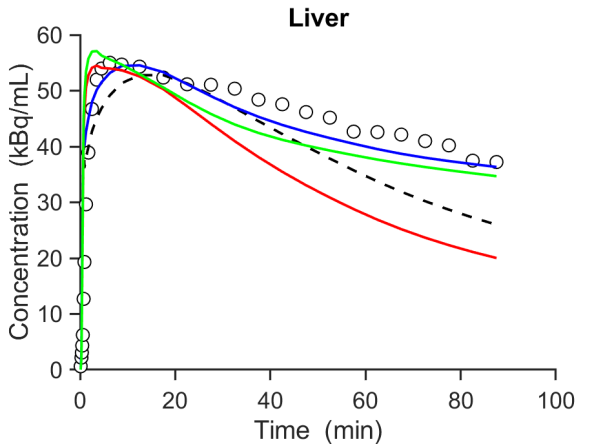


a

b

d


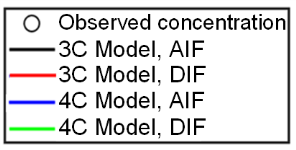

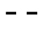

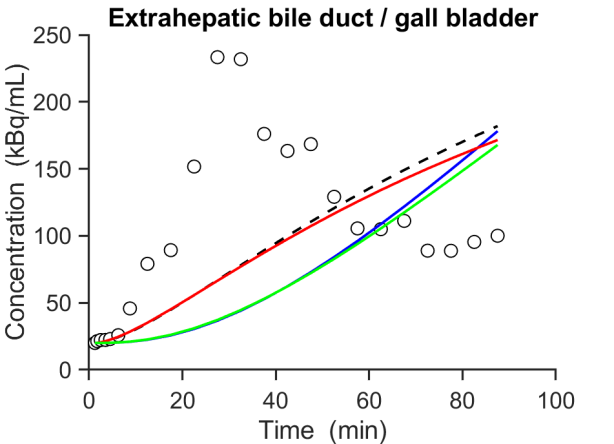

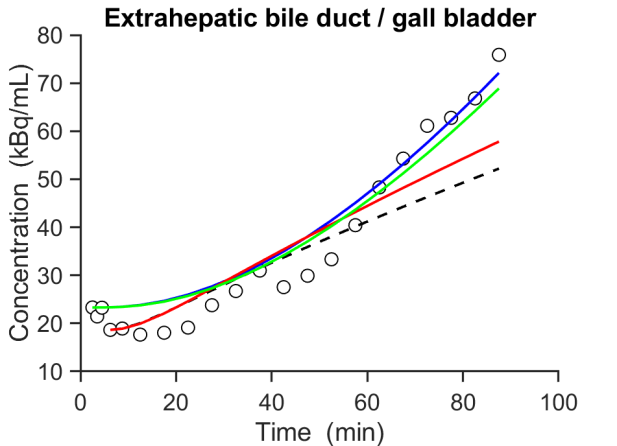


**Supplemental Fig. 1.** Concentration-time profiles of observed data and model predictions for subject p13 in baseline scan (a, b) and in second scan after oral erlotinib (c, d) ([^11^C]erlotinib + erlotinib study).


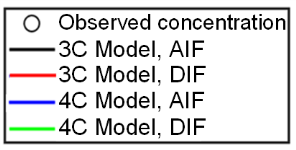

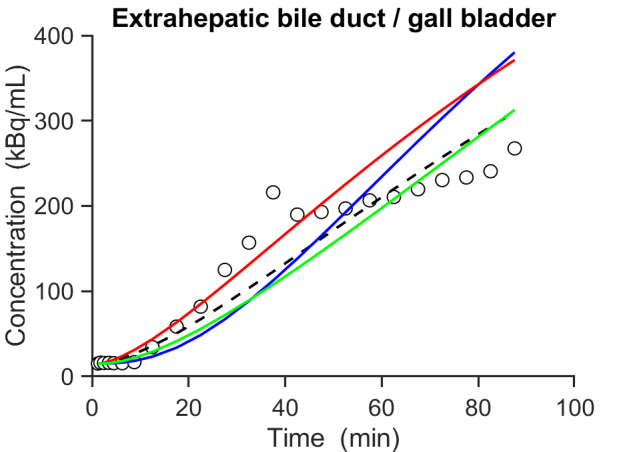

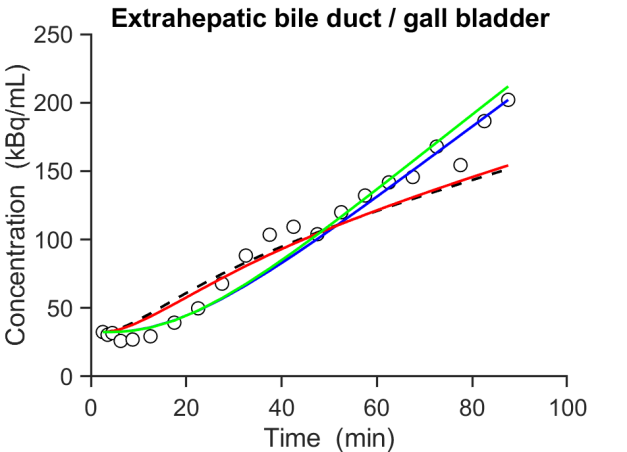

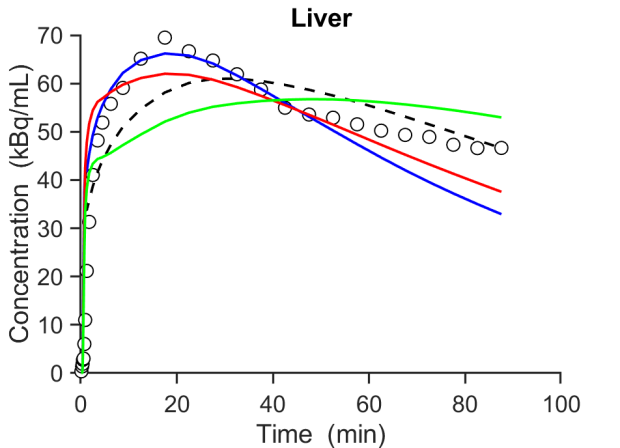

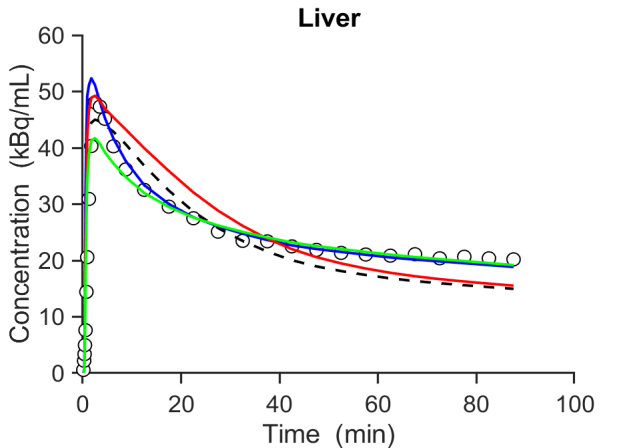


a

c

b

d


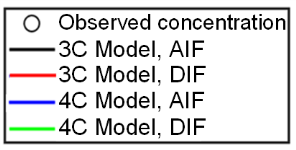

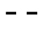


**Supplemental Fig. 2.** Concentration-time profiles of observed data and model predictions for subject p14 in baseline scan (a, b) and in second scan after oral erlotinib (c, d) ([^11^C]erlotinib + erlotinib study).


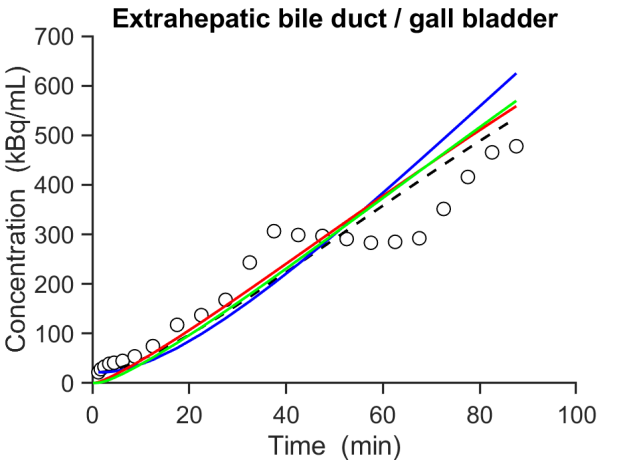

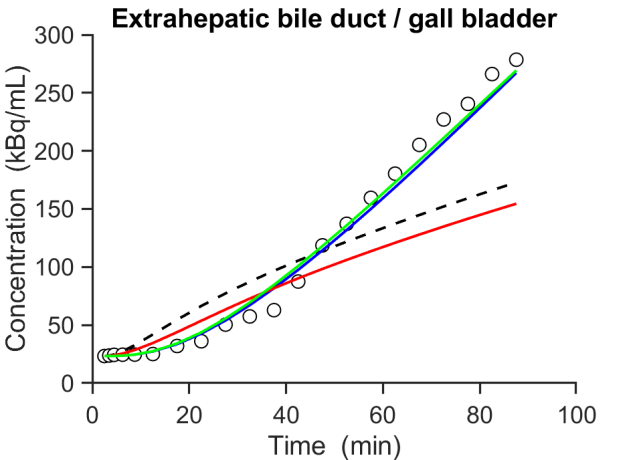

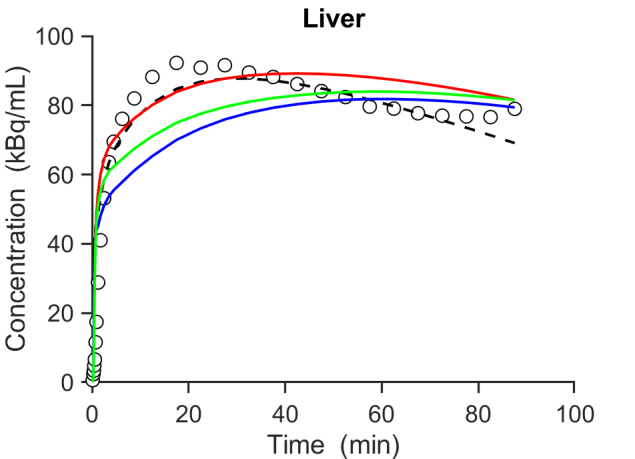

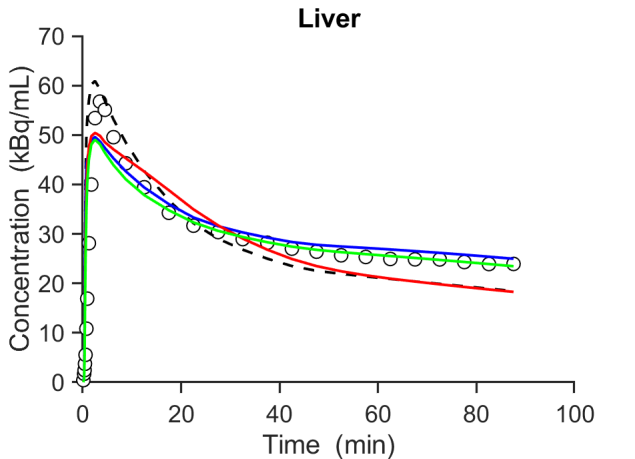


c

a

b

d


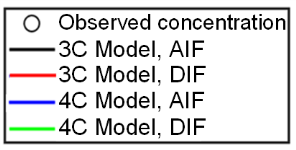

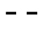


**Supplemental Fig. 3.** Concentration-time profiles of observed data and model predictions for subject p15 in baseline scan (a, b) and in second scan after oral erlotinib (c, d) ([^11^C]erlotinib + erlotinib study).


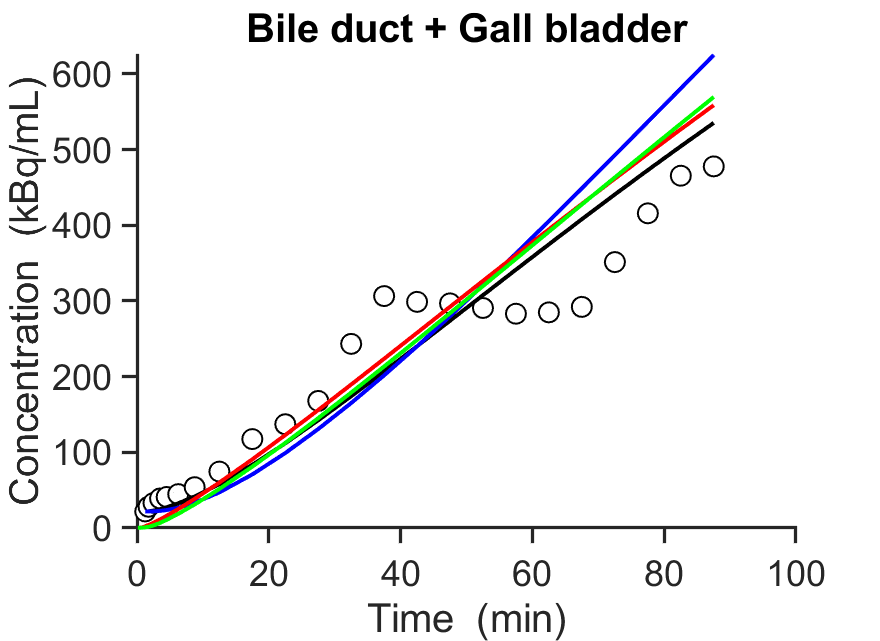

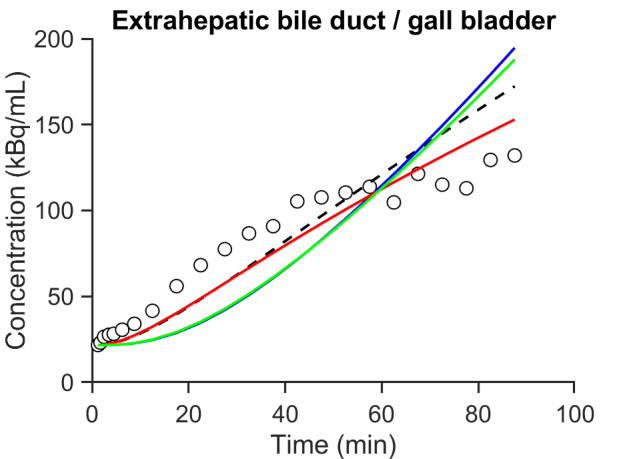

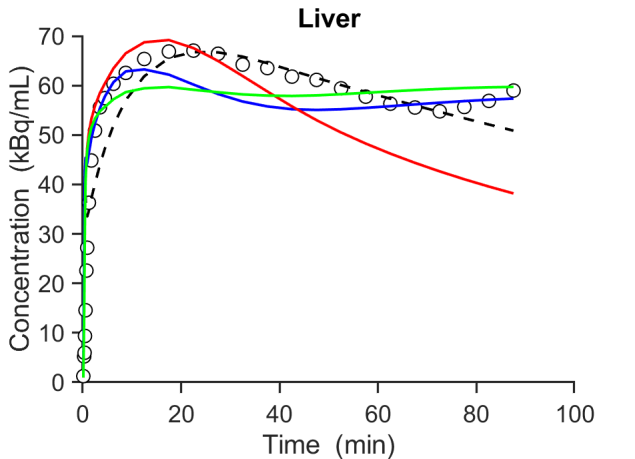

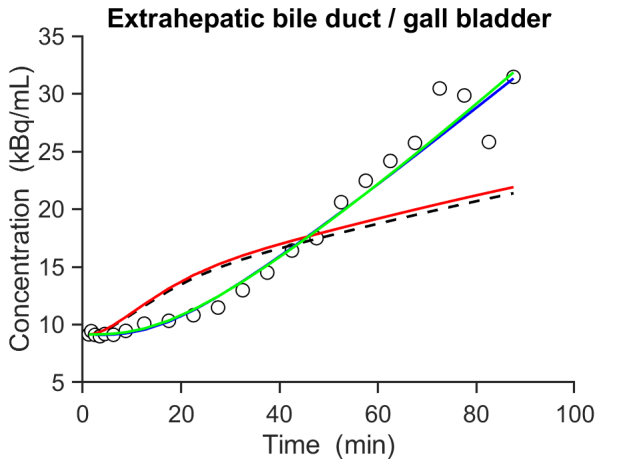

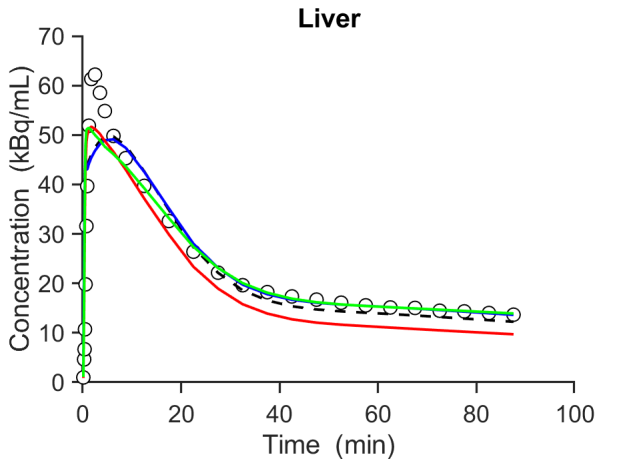


a

c

b

d


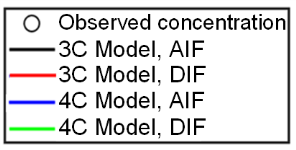

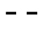


**Supplemental Fig. 4.** Concentration-time profiles of observed data and model predictions for subject p24 in baseline scan (a, b) and in second scan after oral erlotinib (c, d) ([^11^C]erlotinib + erlotinib study).


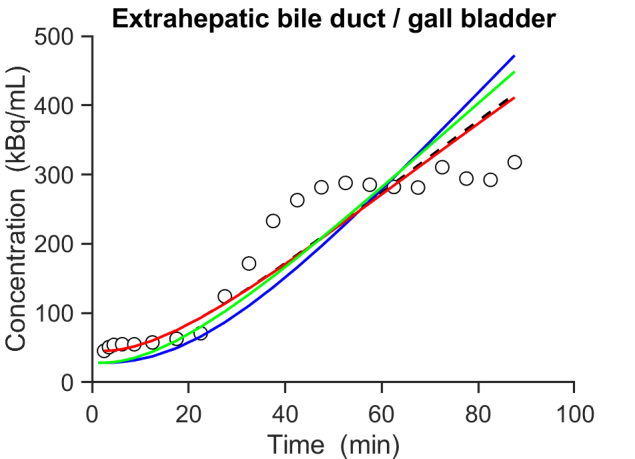

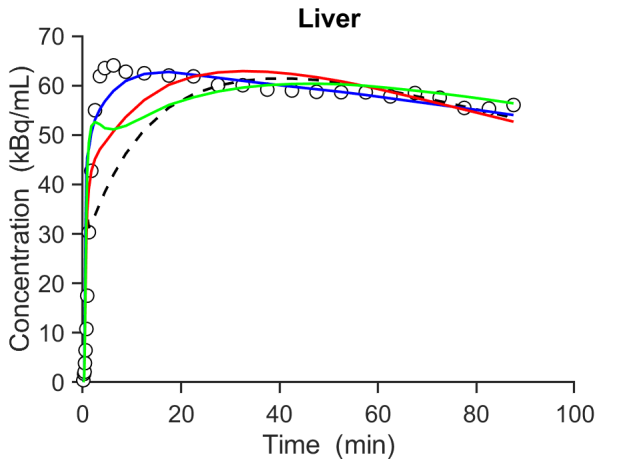

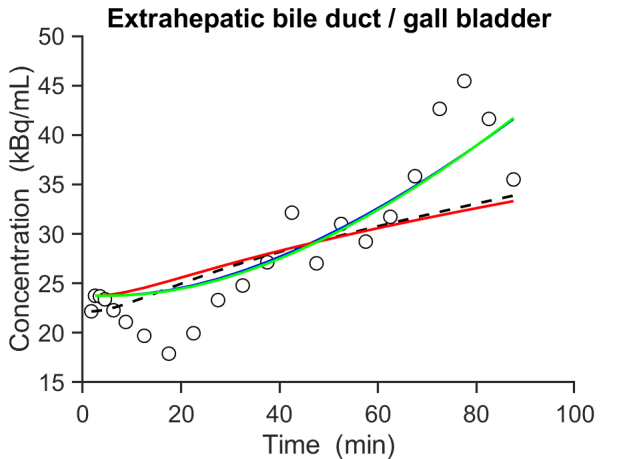

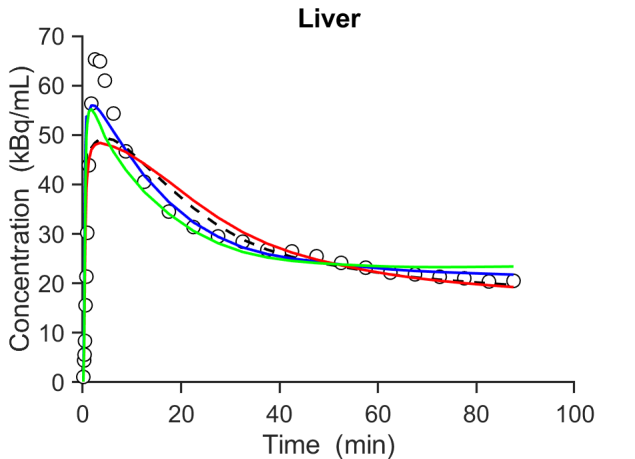

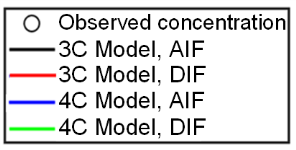

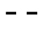


a

c

b

d

**Supplemental Fig. 5.** Concentration-time profiles of observed data and model predictions for subject p30 in baseline scan (a, b) and in second scan after oral erlotinib (c, d) ([^11^C]erlotinib + erlotinib study).

3C Model

4C Model

Arterial Input Function

Dual Input Function


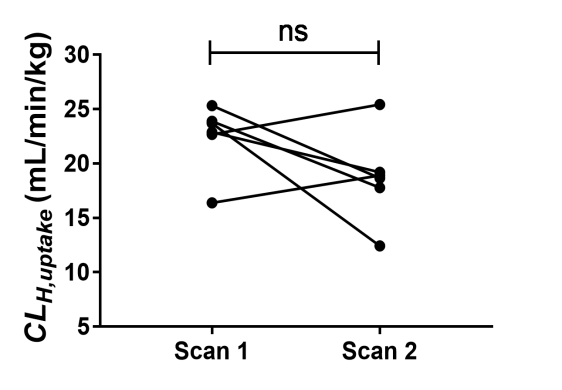

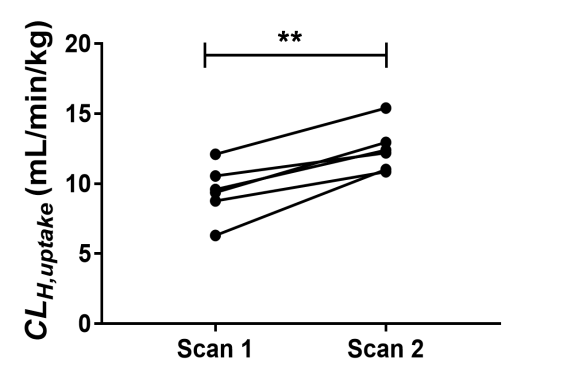

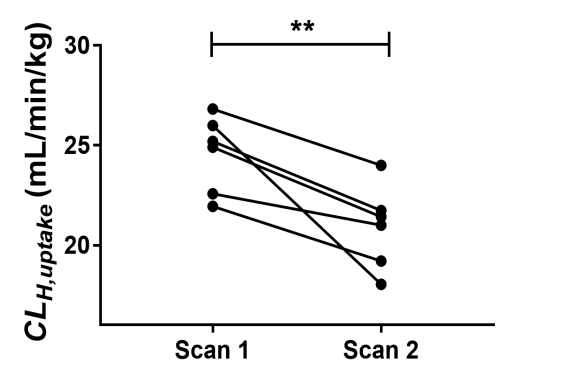

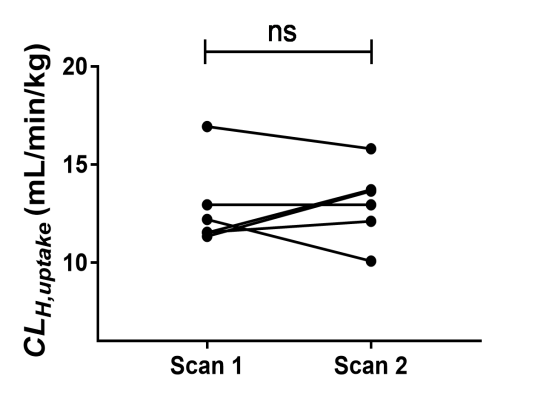


**Supplemental Fig. 6.** Changes in hepatic uptake clearance (*CL_H,uptake_*) from scan 1 to scan 2 for the [^11^C]erlotinib + erlotinib data set. *CL_H,uptake_* was obtained by multiplying the rate constant defining the radiotracer transfer form blood to liver (*k_1_*) by the volume of blood in the liver (0.25 mL of blood/ mL of liver) and dividing by the weight of the individual. ns, not significant; *, p ≤ 0.05; **, p ≤ 0.01, two-tailed paired t-test.

Arterial Input Function

Dual Input Function

3C Model

4C Model


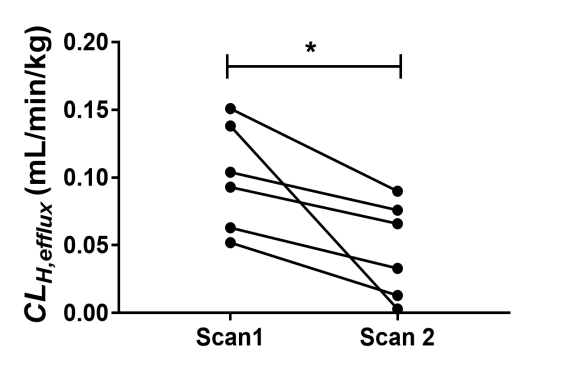

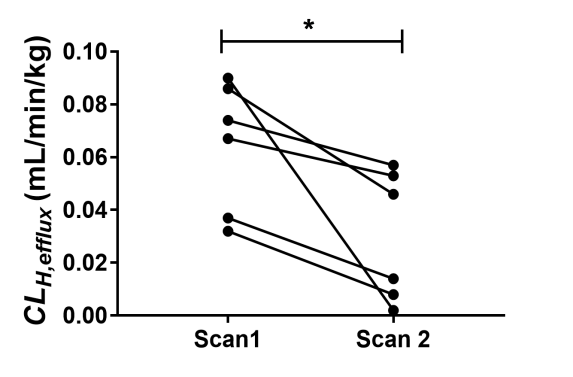

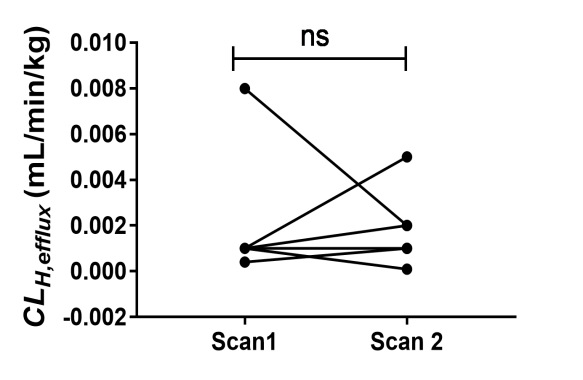

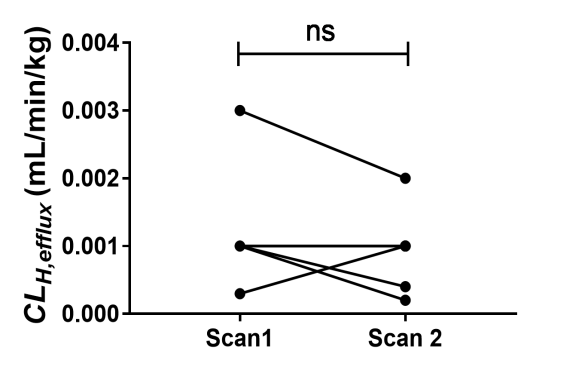


**Supplemental Fig. 7.** Changes in efflux clearance (*CL_H,efflux_*) from scan 1 to scan 2 for the [^11^C]erlotinib + erlotinib data set. *CL_H,efflux_* was obtained by multiplying the rate constant defining the radiotracer transfer from liver to extrahepatic bile duct/gall bladder (*k_3_* in the case of 3C model and *k_5_* for 4C model) by either the volume of the intrahepatic bile duct (0.0032 mL of intrahepatic bile duct/mL of liver) for the 4C model or the volume of the liver tissue for the 3C model, and then dividing by the body weight of the subject to obtain the final clearance. ns, not significant; *, p ≤ 0.05, two-tailed paired t-test.

c


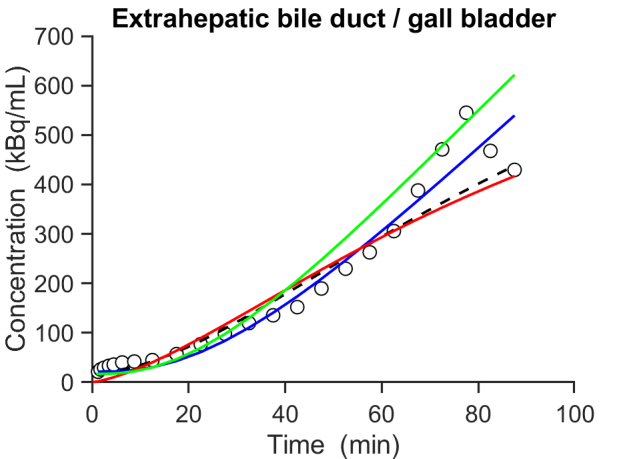

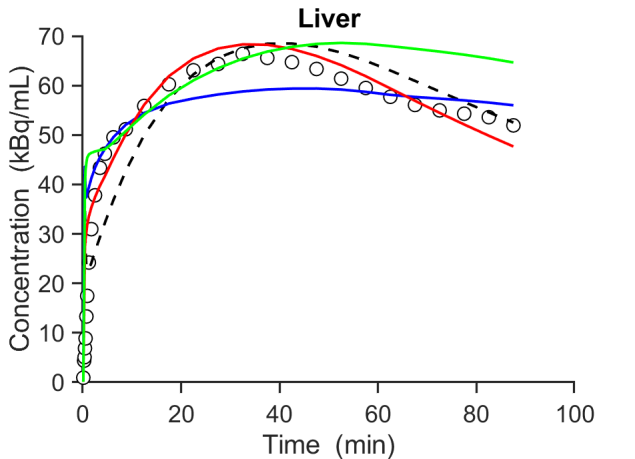

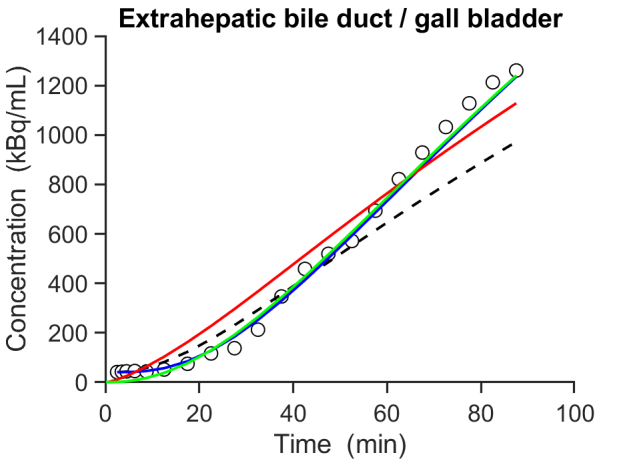

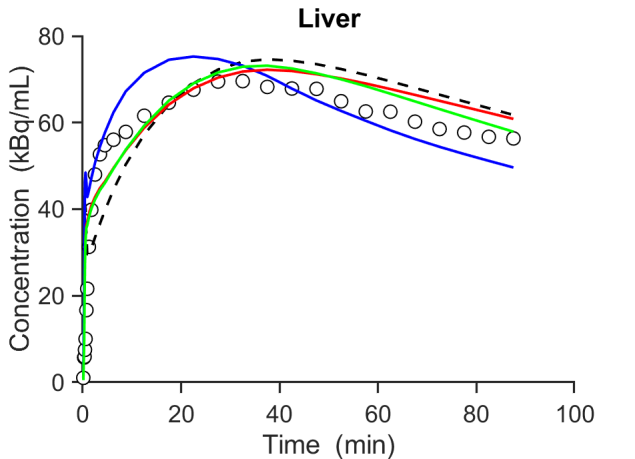

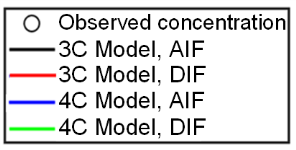

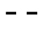


a

b

d

b

**Supplemental Fig. 8.** Concentration-time profiles of observed data and model predictions for subject p37 in baseline scan (a, b) and in second scan after i.v. rifampicin (c, d) ([^11^C]erlotinib + rifampicin study).


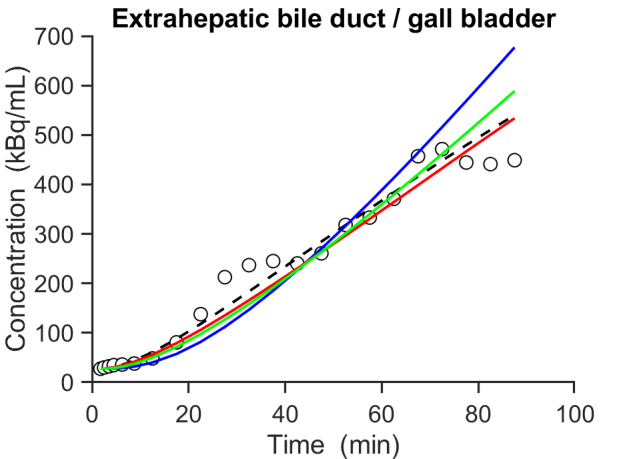

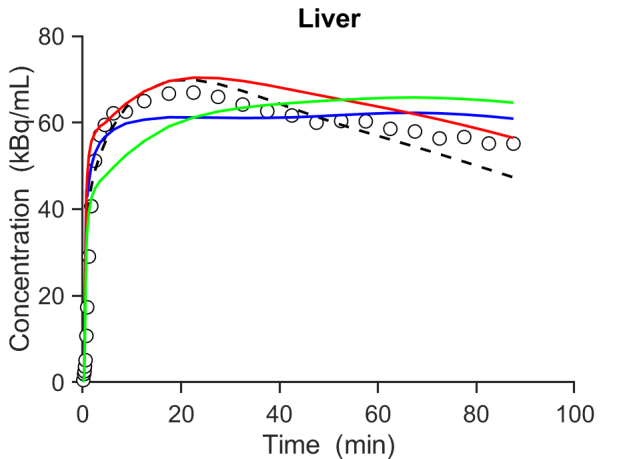

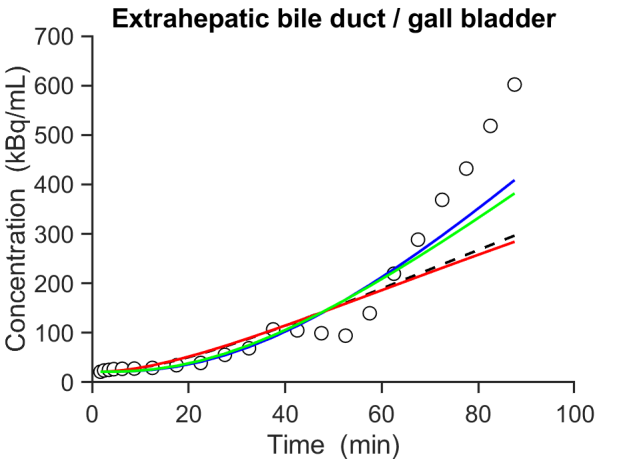

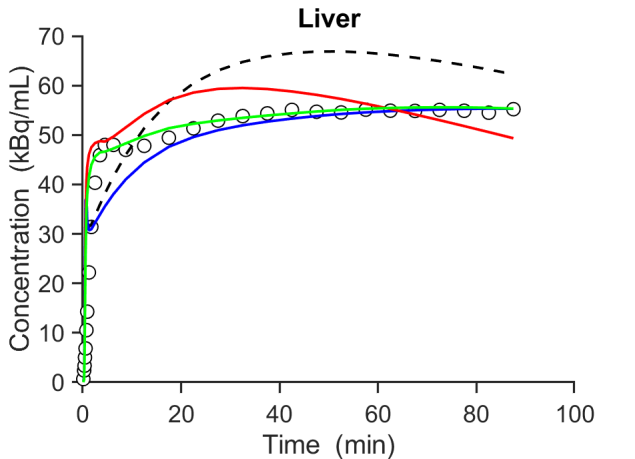

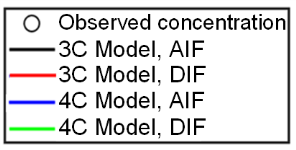

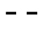


a

c

b

d

**Supplemental Fig. 9.** Concentration-time profiles of observed data and model predictions for subject p38 in baseline scan (a, b) and in second scan after i.v. rifampicin (c, d) ([^11^C]erlotinib + rifampicin study).


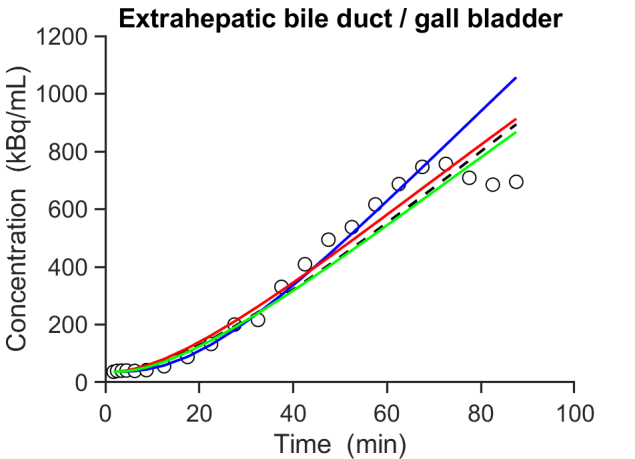

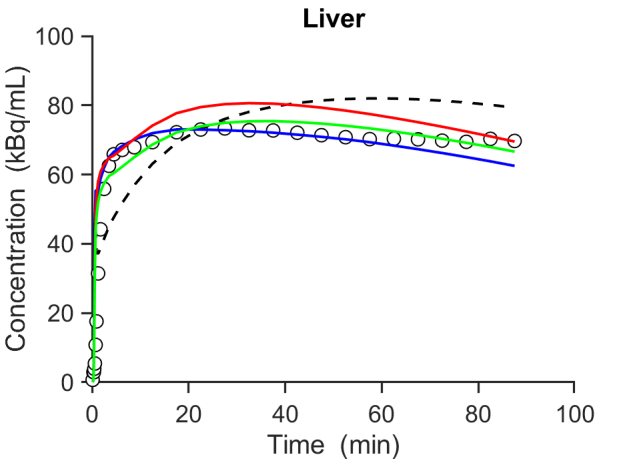

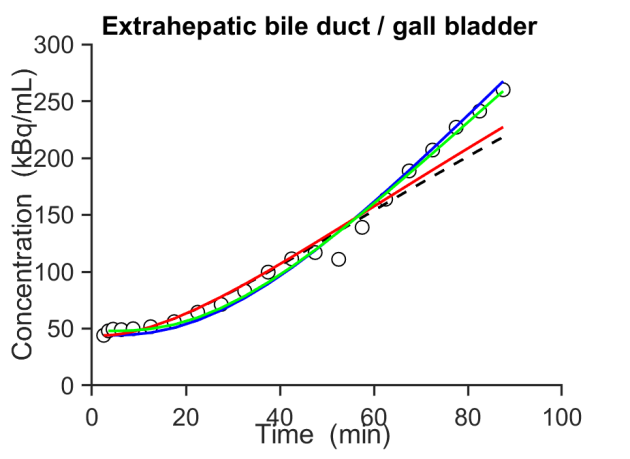

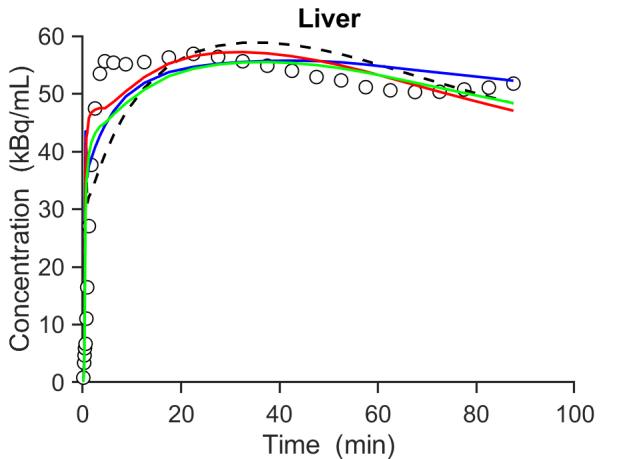

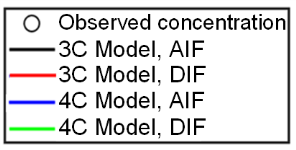

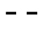


c

a

d

b

**Supplemental Fig. 10.** Concentration-time profiles of observed data and model predictions for subject p39 in baseline scan (a, b) and in second scan after i.v. rifampicin (c, d) ([^11^C]erlotinib + rifampicin study).


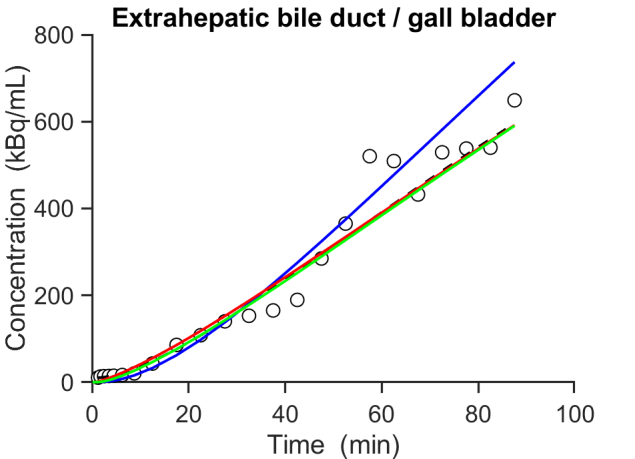

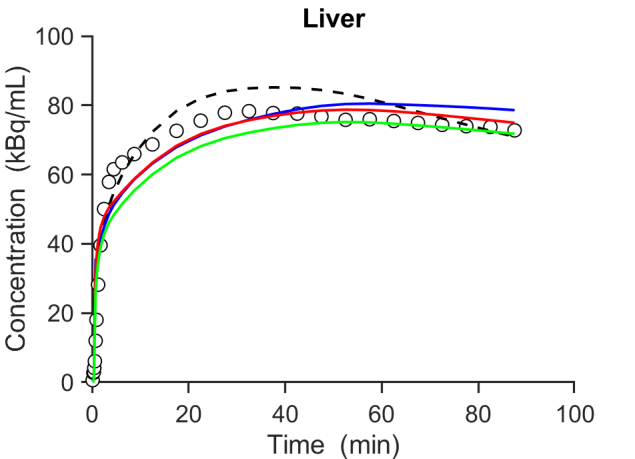

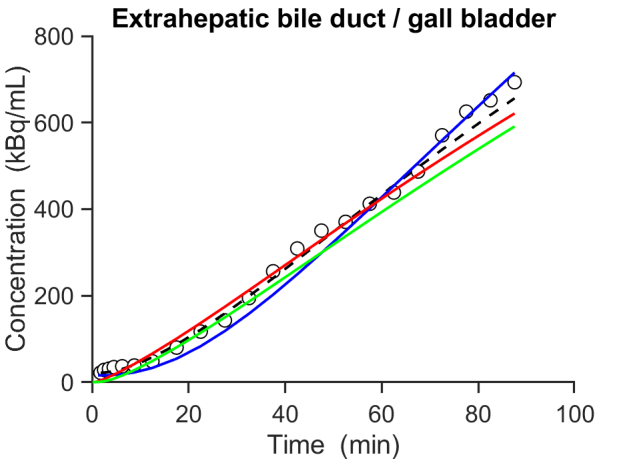

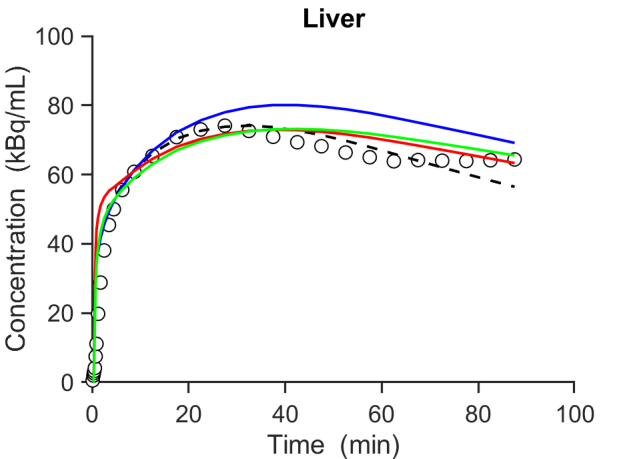

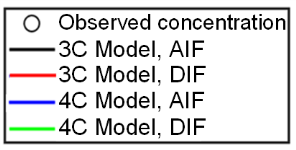

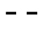


a

c

b

d

**Supplemental Fig. 11.** Concentration-time profiles of observed data and model predictions for subject p41 in baseline scan (a, b) and in second scan after i.v. rifampicin (c, d) ([^11^C]erlotinib + rifampicin study).


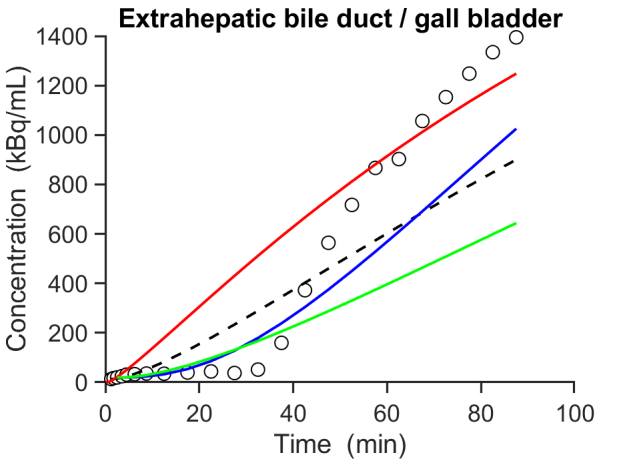

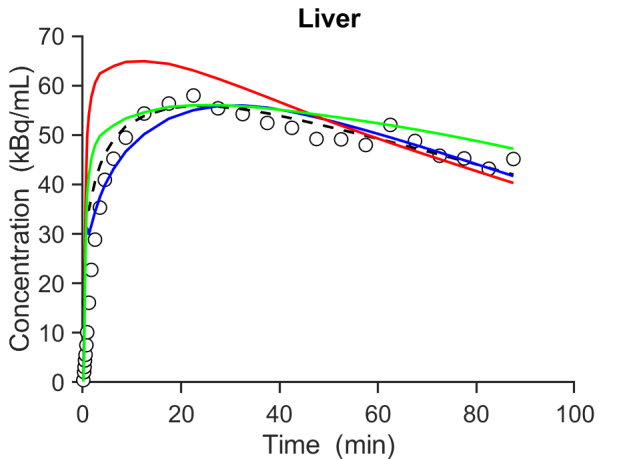

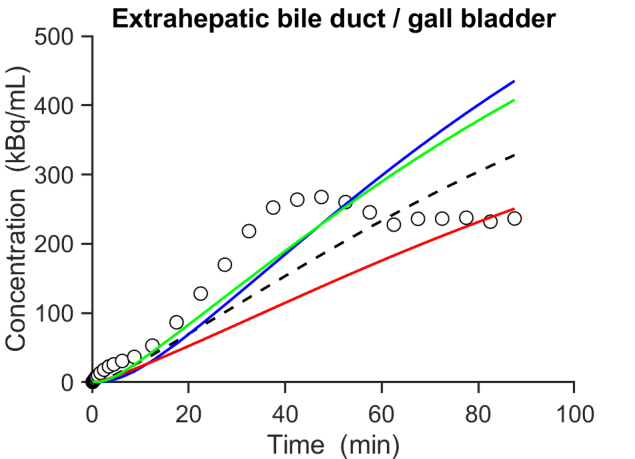

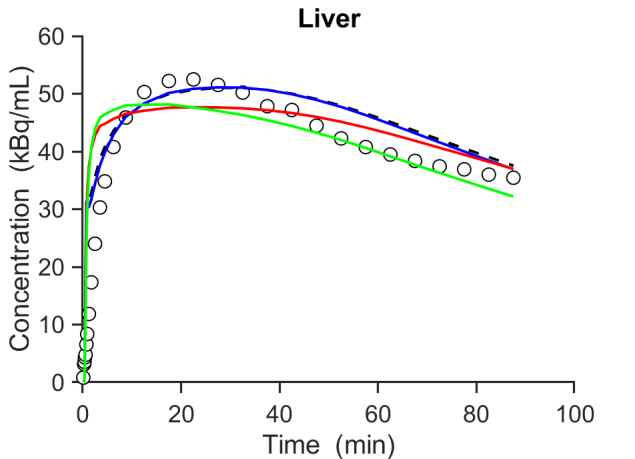

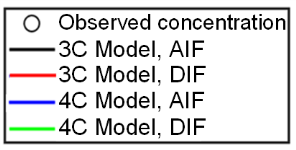

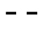


a

c

b

d

**Supplemental Fig. 12.** Concentration-time profiles of observed data and model predictions for subject p42 in baseline scan (a, b) and in second scan after i.v. rifampicin (c, d) ([^11^C]erlotinib + rifampicin study).

3C Model

4C Model

Arterial Input Function

Dual Input Function


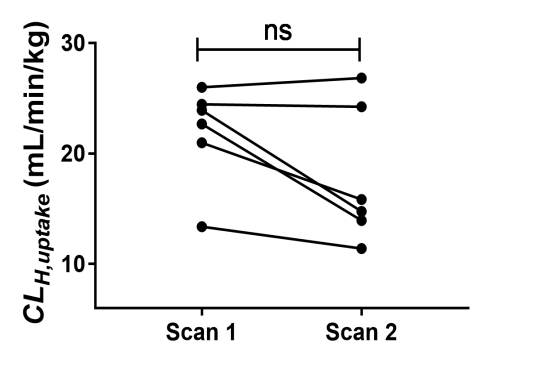

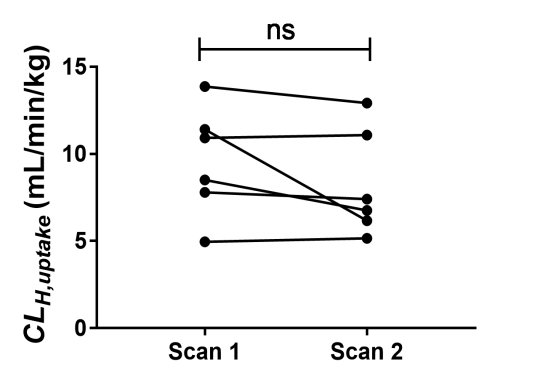

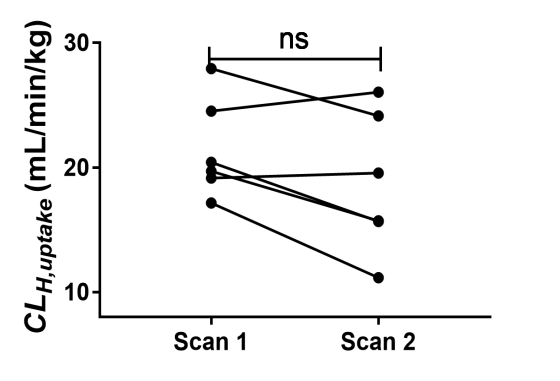

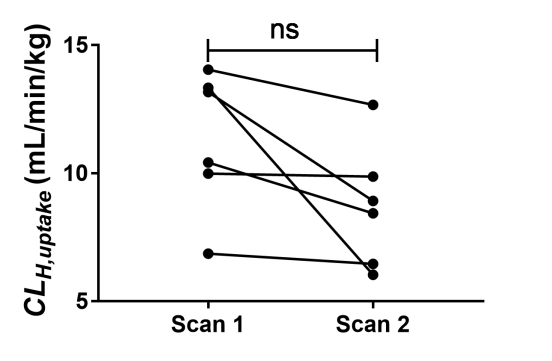


**Supplemental Fig. 13.** Changes in hepatic uptake clearance (*CL_H,uptake_*) from scan 1 to scan 2 for the [^11^C]erlotinib + rifampicin data set. *CL_H,uptake_* was obtained by multiplying the rate constant defining the radiotracer transfer form blood to liver (*k_1_*) by the volume of blood in the liver (0.25 mL of blood/ mL of liver) and dividing by the weight of the individual. ns, not significant, two-tailed paired t-test.

3C Model

4C Model

Arterial Input Function

Dual Input Function


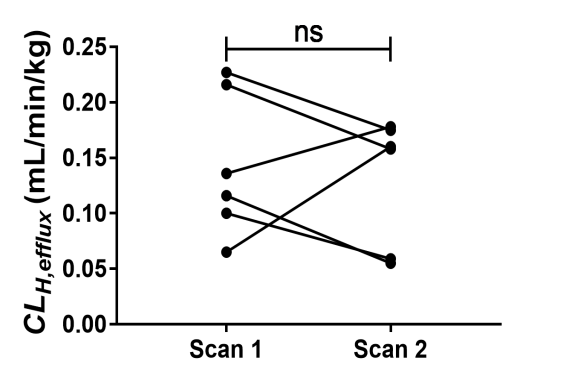

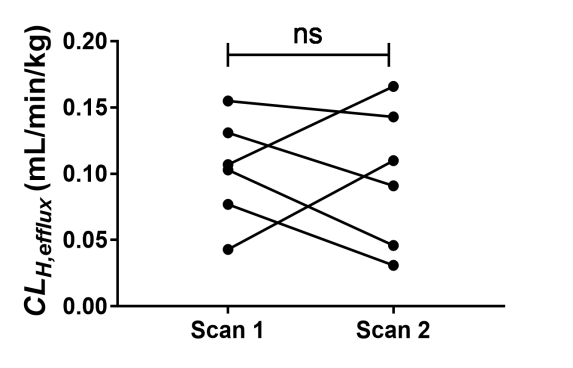

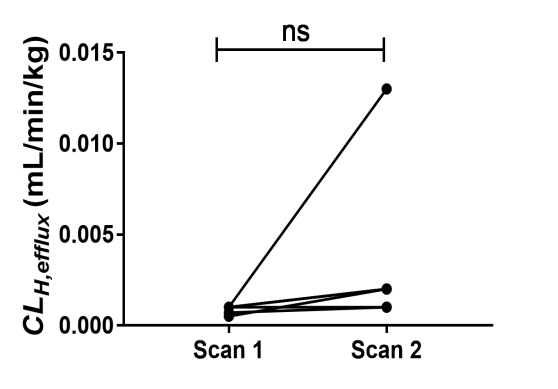

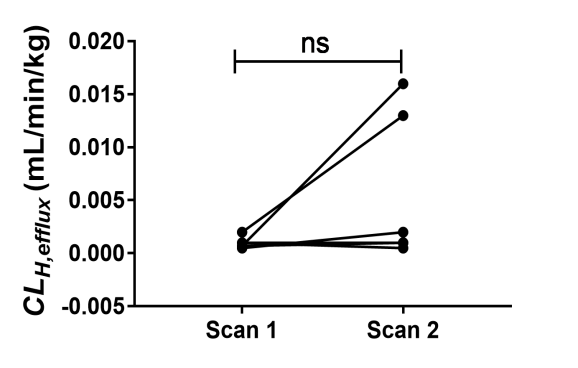


**Supplemental Fig. 14.** Changes in efflux clearance (*CL_H,efflux_*) from scan 1 to scan 2 for the [^11^C]erlotinib + rifampicin data set. *CL_H,efflux_* was obtained by multiplying the rate constant defining the radiotracer transfer from liver to extrahepatic bile duct/gall bladder (*k_3_* in the case of 3C model and *k_5_* for 4C model) by either the volume of the intrahepatic bile duct (0.0032 mL of intrahepatic bile duct/mL of liver) for the 4C model or the volume of the liver tissue for the 3C model, and then dividing by the body weight of the subject to obtain the final clearance. ns, not significant, two-tailed paired t-test.

**
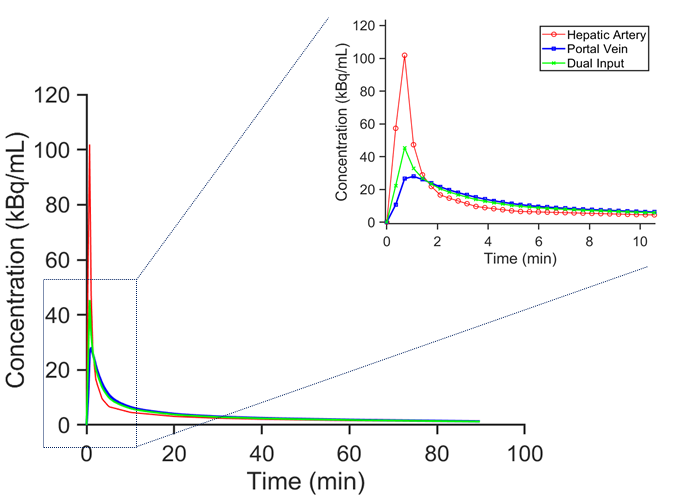
**

**Supplemental Fig. 15.** Representation of the sampled arterial (assumed to equal the hepatic artery), the mathematically-derived portal vein and the dual input functions in one representative subject (p30). The enlarged graph section shows only the first 10 min of the PET scan duration. The value of the estimated β parameter in this subject was 1.050 min.


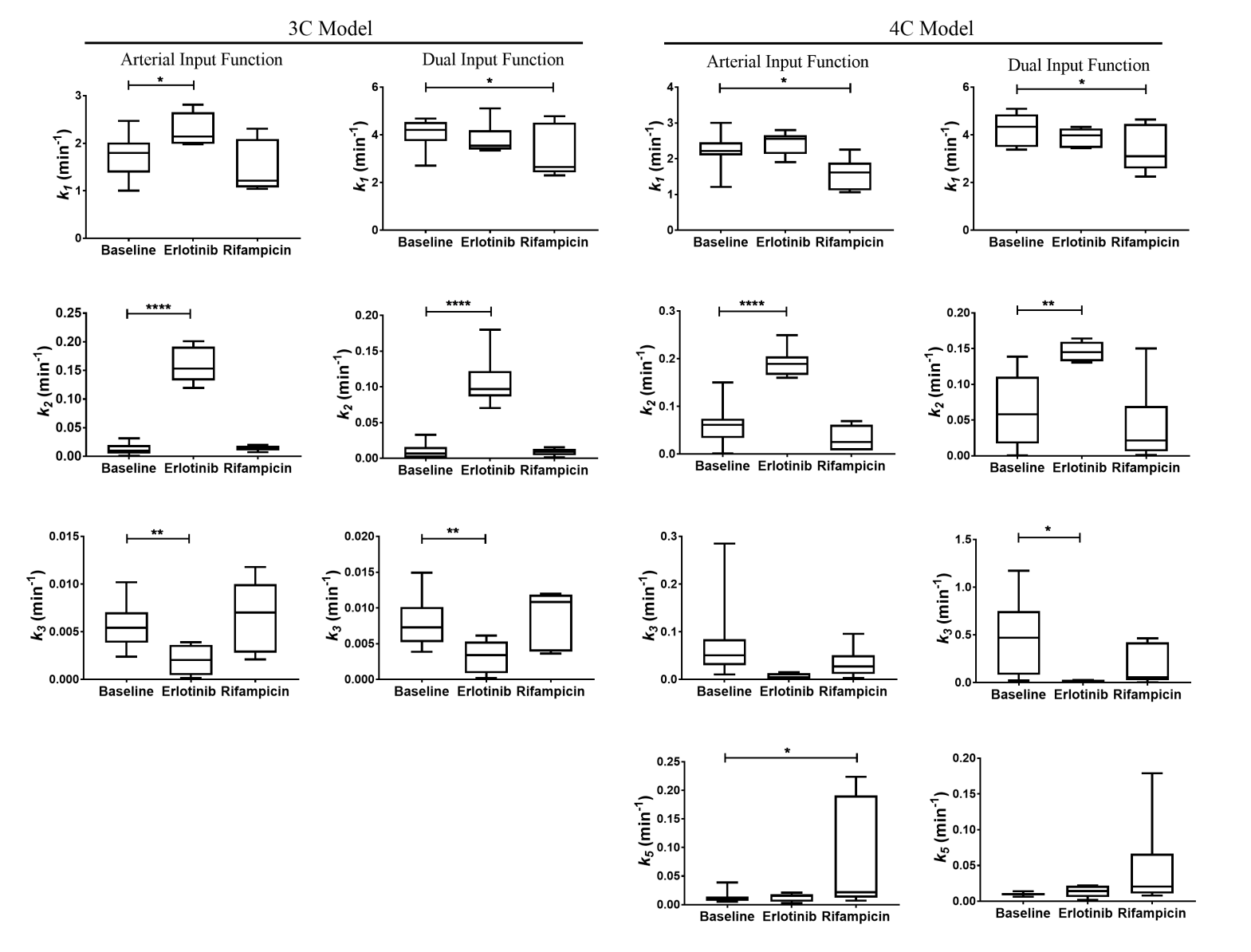
**Supplemental Fig. 16.** Effect of erlotinib or rifampicin administration on the kinetics of [^11^C]erlotinib. “Baseline” refers to the values of all baseline scans (n=12). Whiskers express min to max values of the parameter. *, p ≤ 0.05; **, p ≤ 0.01; ****, p ≤ 0.0001; two-tailed unpaired t-test.


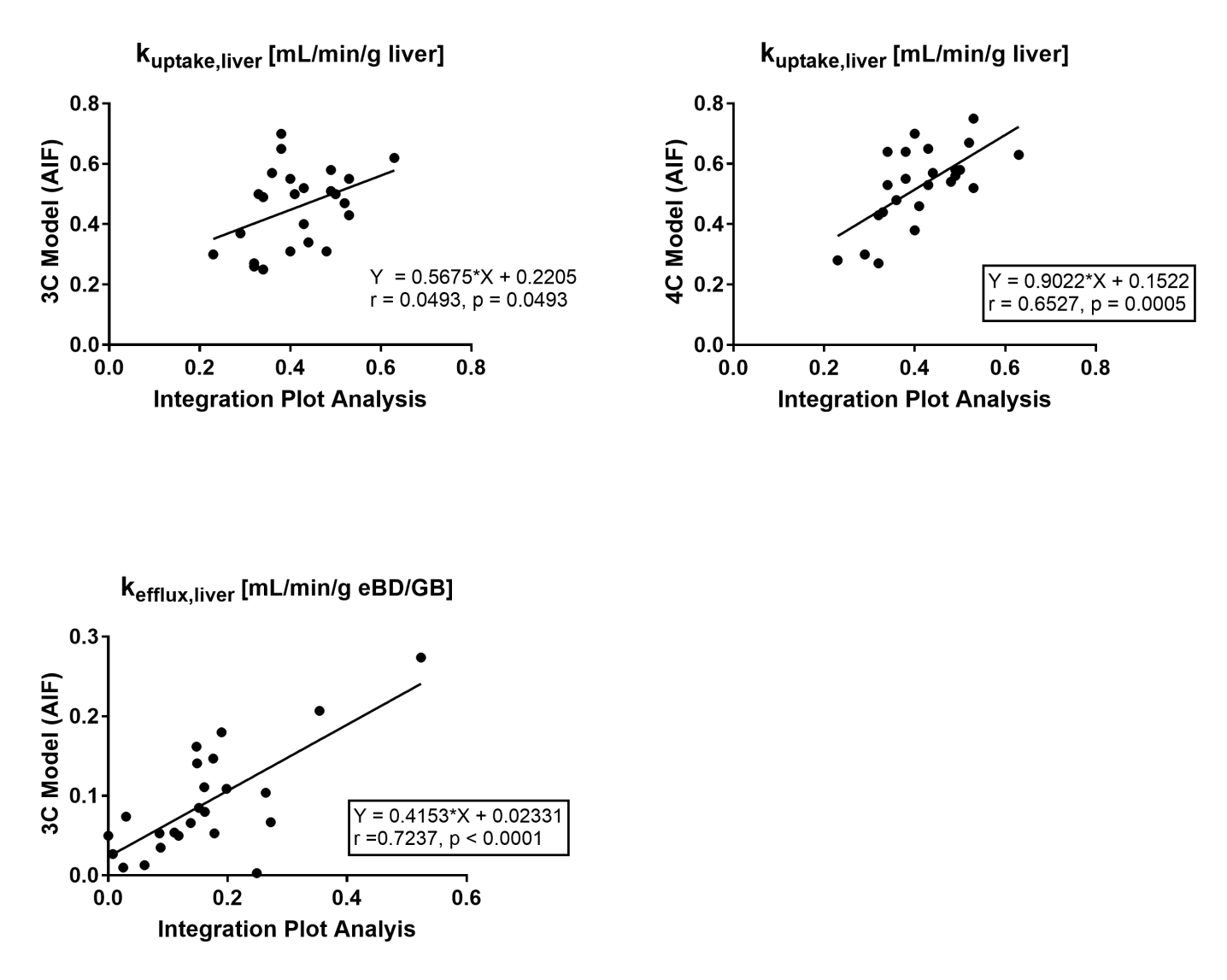


a

c

b

**Supplemental Fig. 17.** Correlations (*r* = Pearson correlation coefficient) between kinetic parameters derived from the 3C and 4C models using AIF and the respective parameters obtained with integration plot analysis (see references 18 and 19 of main text). k_uptake,liver_ represents the radiotracer flow rate from arterial blood into the liver which is calculated by multiplying *k_1_* by the blood volume and dividing by the liver weight. k_efflux,liver_ defines the radiotracer flow rate from the liver into the extrahepatic bile duct/gall bladder and is calculated by multiplying *k_3_* (only from the 3C model since *k_3_* in the 4C model does not have the same meaning) by the liver volume and dividing by the weight of the extrahepatic bile duct/gall bladder (derived from the volume of the PET ROI assuming a density of 1 g/mL). Note that k_uptake,liver_ values shown here are based on AIF and do therefore not represent the transfer of radiotracer from sinusoidal blood into the liver.
